# Supplementary material for: An observational study to examine how cumulative impact zones influence alcohol availability from different types of licensed outlets in an inner London Borough
Source: BMJ Open. 2019 Sep 24;9(9):e027320. doi: 10.1136/bmjopen-2018-027320 (PMC6773415; doi:10.1136/bmjopen-2018-027320)
Supplement: Supplementary data [file bmjopen-2018-027320supp001.pdf]

Supplementary Material

Supplementary Table 1: Area and estimated population for Southwark's three CIZs, the control region and the London Borough of Southwark

	AREA (km^2)	ESTIMATED TOTAL POPULATION (2016)**	
		**Minimum**	**Maximum**
**Bankside**	1.69	15,214	16,018
**Peckham**	0.91	11,226	13,820
**Camberwell**	0.74	6,518	8,474
**Control***	26.47	273,343	278,697
**Southwark**	29.81	311,655	

*The control region relates to the area of Southwark that is not a CIZ
**Southwark's CIZs are non-standard geographies: their boundaries do not align with Output Areas (OA). Output Areas are the lowest level of geography produced across all census topics. The average population in England and Wales on 27 March 2011 was 309 in OAs. Therefore, each CIZ population can only be estimated. The minimum population estimate relates the total population of all OAs that fully fit within the CIZ boundary. The maximum estimate relates the to the total population of all the OAs that fall fully within the CIZ boundary as well as those that are partially within the CIZ boundary. [1]

[1] Office for National Statistics, 2011 Census: Population and Household Estimates for Small Areas in England and Wales, March 2011, [Available from www.ons.gov.uk], 2012

Supplementary Table 2: Number of licence applications and percentage issued in each CIZ and the control group by financial year. Data are stratified by outlet type (five categories).

	2006/07	2007/08	2008/09	2009/10	2010/11	2011/12	2012/13	2013/14	2014/15	2015/16	2016/17	Total
**DRINKING ESTABLISHMENTS**												
**All CIZs**	**21**	**17**	**7**	**6**	**4**	**5**	**11**	**5**	**3**	**5**	**5**	**89**
% Issued	85.7	88.2	100	100	75.0	100	81.8	100	100	100	60.0	88.8
Bankside	**11**	**11**	**5**	**4**	**4**	**5**	**10**	**3**	**2**	**5**	**3**	**63**
% Issued	100	90.9	100	100	75.0	100	80.0	100	100	100	66.7	92.1
Peckham	**3**	**3**	**2**	**2**	**0**	**0**	**1**	**2**	**1**	**0**	**2**	**16**
% Issued	100	100	100	100	-	-	100	100	100	-	50.0	93.8
Camberwell	**7**	**3**	**0**	**0**	**0**	**0**	**0**	**0**	**0**	**0**	**0**	**10**
% Issued	57.1	66.7	-	-	-	-	-	-	-	-	-	60.0
**Control**	**47**	**19**	**11**	**3**	**6**	**8**	**6**	**6**	**15**	**7**	**20**	**148**
% Issued	91.5	73.7	81.8	66.7	83.3	75.0	100	83.3	80.0	85.7	85.0	84.5
**EATERIES**												
**All CIZs**	**18**	**12**	**9**	**15**	**8**	**8**	**14**	**18**	**29**	**21**	**20**	**172**
% Issued	100	91.7	77.8	100	100	100	92.9	88.9	100	95.2	90.0	94.8
Bankside	**15**	**9**	**6**	**10**	**4**	**6**	**9**	**9**	**17**	**16**	**13**	**114**
% Issued	100	88.9	83.3	100	100	100	88.9	88.9	100	93.8	84.6	93.9
Peckham	**1**	**0**	**2**	**3**	**2**	**1**	**5**	**5**	**7**	**5**	**3**	**34**
% Issued	100	0.0	50.0	100	100	100	100	80.0	100	100	100.0	94.1
Camberwell	**2**	**3**	**1**	**2**	**2**	**1**	**0**	**4**	**5**	**0**	**4**	**24**
% Issued	100	100	100	100	100	100	-	100	100	-	100	100
**Control**	**49**	**17**	**15**	**14**	**18**	**19**	**13**	**21**	**19**	**31**	**39**	**255**
% Issued	93.9	94.1	93.3	85.7	94.4	100	100	85.7	84.2	77.4	94.9	91.0
**TAKEAWAYS**												
**All CIZs**	**4**	**1**	**0**	**2**	**4**	**2**	**1**	**3**	**1**	**3**	**11**	**32**
% Issued	100	100	-	50.0	100	100	100	100	100	100	90.9	93.8
Bankside	**3**	**0**	**0**	**1**	**3**	**2**	**1**	**0**	**0**	**2**	**6**	**18**
% Issued	100	-	-	100	100	100	100	-	-	100	100	100
Peckham	**0**	**1**	**0**	**1**	**0**	**0**	**0**	**1**	**1**	**1**	**4**	**9**
% Issued	-	100	-	0	-	-	-	100	100	100	75.0	77.8
Camberwell	**1**	**0**	**0**	**0**	**1**	**0**	**0**	**2**	**0**	**0**	**1**	**5**
% Issued	100	-	-	-	100	-	-	100	-	-	100	100
**Control**	**19**	**6**	**2**	**1**	**1**	**6**	**1**	**4**	**3**	**5**	**7**	**55**
% Issued	100	83.3	100	100	100	66.7	100	100	100	100	71.4	90.9
**OFF-SALES**												
**All CIZs**	**14**	**5**	**6**	**2**	**7**	**6**	**4**	**11**	**4**	**4**	**5**	**68**
% Issued	100	80.0	66.7	100	85.7	66.7	100	90.9	100	100	100	89.7
Bankside	**7**	**3**	**5**	**1**	**5**	**1**	**1**	**5**	**4**	**3**	**4**	**39**
% Issued	100	100	60.0	100	100	100	100	100	100	100	100	94.9
Peckham	**5**	**1**	**0**	**1**	**2**	**2**	**3**	**2**	**0**	**1**	**1**	**18**
% Issued	100.0	-	-	100	50.0	-	100	50.0	-	100	100	72.2
Camberwell	**2**	**1**	**1**	**0**	**0**	**3**	**0**	**4**	**0**	**0**	**0**	**11**
% Issued	100	100	100	-	-	100	-	100	-	-	-	100

**Control**	**40**	**18**	**15**	**11**	**9**	**17**	**10**	**18**	**16**	**16**	**10**	**180**
% Issued	92.5	94.4	86.7	81.8	100	100	100	100	81.3	68.8	70.0	89.4
**OTHER OUTLETS**												
**All CIZs**	**9**	**10**	**12**	**12**	**12**	**6**	**10**	**9**	**8**	**21**	**11**	**120**
% Issued	88.9	100	83.3	83.3	91.7	83.3	100	66.7	87.5	85.7	72.7	85.8
Bankside	**7**	**10**	**10**	**12**	**9**	**5**	**7**	**6**	**7**	**15**	**9**	**97**
% Issued	85.7	100	90.0	83.3	100	80.0	100	66.7	100	80.0	77.8	87.6
Peckham	**1**	**0**	**2**	**0**	**2**	**1**	**3**	**3**	**0**	**5**	**1**	**18**
% Issued	100	-	50.0	0.0	50.0	100	100	66.7	-	100	0.0	77.8
Camberwell	**1**	**0**	**0**	**0**	**1**	**0**	**0**	**0**	**1**	**1**	**1**	**5**
% Issued	100	-	-	-	100	-	-	-	0.0	100	100	80.0
**Control**	**18**	**6**	**5**	**12**	**8**	**10**	**10**	**11**	**19**	**20**	**16**	**135**
% Issued	94.4	100	60.0	83.3	100	100	100	100	78.9	80.0	62.5	85.9
