# Supplementary material for: An observational study to examine how cumulative impact zones influence alcohol availability from different types of licensed outlets in an inner London Borough
Source: BMJ Open. 2019 Sep 24;9(9):e027320. doi: 10.1136/bmjopen-2018-027320 (PMC6773415; doi:10.1136/bmjopen-2018-027320)
Supplement: Author's manuscript [file bmjopen-2018-027320.draft_revisions.pdf]

BMJ Open

BMJ Open is committed to open peer review. As part of this commitment we make the peer review history of every article we publish publicly available.

When an article is published we post the peer reviewers' comments and the authors' responses online. We also post the versions of the paper that were used during peer review. These are the versions that the peer review comments apply to.

The versions of the paper that follow are the versions that were submitted during the peer review process. They are not the versions of record or the final published versions. They should not be cited or distributed as the published version of this manuscript.

BMJ Open is an open access journal and the full, final, typeset and author-corrected version of record of the manuscript is available on our site with no access controls, subscription charges or pay-per-view fees (http://bmjopen.bmj.com).

If you have any questions on BMJ Open's open peer review process please email info.bmjopen@bmj.com

BMJ Open

An observational study to examine how Cumulative Impact Zones influence alcohol availability from different types of licensed outlets in an inner London Borough

Journal:	*BMJ Open*
Manuscript ID	bmjopen-2018-027320
Article Type:	Research
Date Submitted by the Author:	16-Oct-2018
Complete List of Authors:	Sharpe, Carolyn; Imperial College London School of Public Health, Department of Primary Care and Public Health; Southwark Council, Public Health Directorate<br>Poots, Alan; Imperial College London, NIHR CLARHC Northwest London; Independent Researcher<br>Watt, Hilary; Imperial College London, Primary Care and Public Health<br>Franklin, David; Southwark Council, Licensing<br>Pinder, Richard; Imperial College London School of Public Health, Department of Primary Care and Public Health; Southwark Council, Public Health Directorate
Keywords:	PUBLIC HEALTH, Cumulative Impact Zone, Alcohol Licensing, Alcohol Availability

SCHOLARONE™
Manuscripts

For peer review only

An observational study to examine how Cumulative Impact Zones influence alcohol availability from different types of licensed outlets in an inner London Borough

Authors:

C A Sharpe, Policy Officer, Imperial College London & Southwark Council[1,2]
carolyn.sharpe1@nhs.net – *corresponding & first author*

Dr A J Poots, Principal Information Analyst, Imperial College London[4,a]

H Watt, Consultant Statistician & Teaching Fellow, Imperial College London[1]

D Franklin, Head of Licensing, Southwark Council[3]

Dr R J Pinder, Consultant in Public Health, Imperial College London & Southwark Council[1,2]

Affiliations at the time of study:

1. Department of Primary Care and Public Health, School of Public Health, Imperial College London, Reynolds Building, St Dunstans Road, London, W6 8RP, United Kingdom

2. Public Health Directorate, Southwark Council, 1st Floor, Hub 1, PO Box 64529, London, SE1P 5LX, United Kingdom

3. Licensing Team, Southwark Council, 3rd Floor, Hub 1, PO Box 64529, London, SE1P 5LX, United Kingdom

4. NIHR CLARHC Northwest London, Imperial College London, Floor 4, Lift Bank D, Chelsea and Westminster Hospital, 369 Fulham Road, London, SW10 9NH, United Kingdom

Current affiliations:

a) Independent researcher

Word Count: 2944 words
(4000 word limit)

Abstract (284 / 300 words)

OBJECTIVES: Cumulative Impact Zones (CIZs) are a widely implemented local policy intended to restrict alcohol availability in areas proliferated with licensed outlets. Limited previous research has questioned their effectiveness and suggested they may play a more nuanced role in shaping local alcohol environments. This study aims to evaluate the effect of CIZ implementation on five outlet type categories, relative to a control region.

DESIGN: A quantitative observational study.

SETTING: The London Borough of Southwark, an inner London borough, which currently enforces three CIZs.

POPULATION: Licence applications received by Southwark Council's Licensing Authority between 1 April 2006 and 31 March 2017 (N = 1254).

INTERVENTIONS: CIZ implementation.

PRIMARY OUTCOME MEASURES: Five outlet types were categorised and evaluated: Drinking Establishments, Eateries, Takeaways, Off Sales, and Other Outlets. Primary outcome measures were the number of applications received and the number of licences issued. These were analysed using Poisson regression of counts over time.

RESULTS: Across all CIZs, implementation was associated with an increase in the number of eateries (IRR = 1.58, 95% CI: 1.02 – 2.52, P = 0.04) and takeaways (IRR = 3.89, 95% CI: 1.32 – 11.49, P = 0.01), compared to the control area. There was no discernible effect on remaining outlet types. Disaggregating by area indicated a 10-fold increase in the number of new eateries in Peckham CIZ (IRR = 10.38, 95% CI: 1.39 – 77.66, P = 0.02) and a four-fold increase in the number of licensed takeaways in Bankside CIZ (IRR = 4.38, 95% CI: 1.20 – 15.91, P = 0.03), compared to the control area.

CONCLUSIONS: Cumulative Impact Zones may be useful as policy levers to shape local alcohol environments to support the licensing goals of specific geographical areas and diversify the night-time economy.

For peer review only - http://bmjopen.bmj.com/site/about/guidelines.xhtml

Article Summary

Strengths and limitations of this study

- This study provides the first quantitative evaluation of CIZs that disaggregates outlet type into five discrete categories.
- This research provides deeper insight in to the nature of alcohol exposure in different locations within an urban centre and therefore provides an evidence base from which local policy can be developed to support local licensing goals which may include diversification of the night-time economy.
- This study shows that descriptive analyses of alcohol control policies (using absolute numbers) are insufficient, and provides a robust statistical approach that can be employed in other urban settings.
- Although substantial steps were taken to assure the reproducibility of the results, the categorisation of outlet type into five discrete categories involved qualitative assessment and there remains a risk of subjectivity.
- Necessary stratification of the dataset rendered some strata with very few (or in some cases zero) counts, thereby constraining statistical power.

Key words

Public Health, Cumulative Impact Zone, Alcohol Licensing, Alcohol Availability

For peer review only

For peer review only - http://bmjopen.bmj.com/site/about/guidelines.xhtml

Main Text

INTRODUCTION

Alcohol harm and opportunities to control availability

Alcohol occupies a prominent role in modern society. It is a commodity used by many, across social strata, as a relaxant and a means of enjoyment [1]. The misuse of alcohol leads to increased morbidity and mortality. In England in 2016, it was estimated that almost 13 million adults drank at levels that increased their risk of harm. [2] Among those aged 15 to 49, alcohol has become the leading risk factor for ill-health, early mortality, and disability. [3]

Outlet spatial density is positively associated with consumption. [4] [5] Policies to restrict the physical availability of alcohol are well supported in the literature. [6] However, such evidence is based on aggregate studies that consider all alcohol-outlet types as equal. At best, outlets have been categorised as either on or off-sales. On-sales of alcohol refer to venues such as pubs, nightclubs and restaurants where alcohol is purchased to be consumed on the premises. Off-sales of alcohol refer to premises such as off-licenses and supermarkets from which alcohol is purchased to be consumed away from the retailer's premises. [7] Although greater association has been found between off-sales and alcohol-related hospitalisations and deaths than with on-sales, [8] both on- and off-licences cover a broad range of establishments. Such aggregate studies remain a crude way to evaluate alcohol availability. [9]

A full description of the licensing process in England and Wales, as defined by the Licensing Act 2003, [10] is outlined in our previous paper. [11] In summary, Revised Guidance issued under section 182 of the Licensing Act 2003 enables local licensing authorities (LAs) to address the cumulative impact of a concentration of licensed premises. [12] Through the establishment of Cumulative Impact Zones (CIZ) local LAs can better control the number and type of venues in areas where the current saturation of licensed premises is considered likely to undermine the licensing objectives (Box 1). [12]

Box 1: The licensing objectives, in England and Wales, as defined by the Licensing Act 2003 are:

a) The prevention of crime and disorder;
b) Public safety;
c) The prevention of public nuisance; and
d) The protection of children from harm.

For peer review only

Current evidence relating to CIZs

In 2016 there were 215 CIZs established across 106 LAs [13]. Despite their widespread establishment, there is a scarcity of literature evaluating their impact. [14] CIZs are often perceived as a policy lever to limit alcohol availability in areas of high outlet density. [15] However, previous work undertaken in the London Borough of Southwark found no evidence that CIZ establishment reduced the number of licence applications or issued licenses across its three policy areas combined, when compared to a control region. On analysing one CIZ area in isolation, establishment was associated with a statistically significant 133% increase in successful applications. [11]

Qualitative research, conducted in 2016, investigating the drivers behind CIZ implementation has suggested them to be more nuanced than simply restricting outlet density. [16] Participants described how CIZs have evolved to encourage local regeneration goals. CIZs tended not to be established to cap the number of licenced outlets, rather they are viewed as a tool to control the temporal availability of alcohol and even to encourage a certain type of application e.g. from arts, food and coffee led establishments as opposed to 'vertical drinking' bars.

Plans to diversify night-time economies and offer alternatives to alcohol-led activities are emerging. In April 2017, the Mayor of London published guidance for culture and the night-time economy in the capital and identified diversification and inclusiveness as key areas of focus. Through encouraging leisure and cultural venues e.g.: late night markets, museums, shops, cafes, theatres, and fairs, urban centres hope to a wider clientele. Examples of which include non-drinkers, older people, families and those with a disability. [17]

Gaps in the literature

A key research gap exists around the assumption that all on- and off-licensed outlets are treated as equal. [9] Although aggregate studies may show an association between outlet density and harm, they are of limited use to policy makers. Interventions need to be targeted towards outlets that cause the most harm. Therefore, through stratifying by outlet type, we aim to understand how CIZ establishment affects the number of licence applications and issued licences for different outlet types. Qualitative evidence indicates that CIZs can facilitate the proliferation of certain types of premises, thereby diversifying local night-time economies. [16] We therefore propose a quantitative methodology to test for this effect.

For peer review only - http://bmjopen.bmj.com/site/about/guidelines.xhtml

Southwark's three cumulative impact zones

Southwark currently enforces three CIZs; Camberwell CIZ and Peckham CIZ were both established 05 November 2008. Bankside CIZ was established a year later on 05 November 2009. The implementation of each CIZ was to achieve a unique objective. (Franklin D. Head of Licensing, Southwark Council. Personal communication. 17 July 2018) With almost 70% of all CIZ licence applications falling within Bankside alone, the intervention aimed to tackle binge drinking-related issues. The Bankside CIZ policy applies to all venue types except for theatres and cinemas. Peckham and Camberwell CIZs were established to tackle late-night anti-social behaviour and street drinking respectively. Notably, food-led establishments, such as restaurants and cafes, are exempt from both CIZ policies.

For peer review only

METHODS

Data collection and collation

A comprehensive alcohol licensing data set of all applications made within Southwark between 1 April 2006 and 31 March 2017 was obtained from Authority Public Protection (APP); a database used by the council to manage licence applications. [18] Geographical information software (GIS) [19] was used to assign each outlet application an easting and northing. These coordinates were then used to identify applications within each CIZ region and the control group. The issue date was used as a proxy that applications were successful. All applications without an issue date were categorised as having been rejected or withdrawn. An outlet type field was obtained from APP containing 100 different entries for outlet type. This field was aggregated into five mutually-exclusive categories; drinking establishments, eateries, takeaways, off-sales, and other outlets. To ensure reproducibility of the results, two researchers completed the categorisation process independently, before a final list was agreed. The other outlet category was used as a 'catch-all' for outlets that could not be easily categorised. This category contained a range of venues including florists, boats and schools. Licence applications were grouped by financial year (April to the following March) starting from 2006/07. An indicator variable for the intervention was fitted, to indicate which time periods in which regions had a CIZ in operation. The three CIZs were established on 5 November in each of the respective years. We therefore grouped applications in 365-day periods relative to the date of CIZ establishment.

Statistical analyses

Poisson regression [20] was employed to model the effect of CIZ establishment (indicator variable) on the number of applications and of licenses issued (outcome rates of counts). We adjusted for financial yearly trend, across all regions tested, and for geographical area, including both in the model as categorical adjustment variables; financial yearly trend did not fit well enough to a linear model for that to be a viable alternative. An adjustment was made to accommodate for the CIZs being established mid-financial year. Statistical analyses were executed using STATA 13.0. [21] Incidence rate ratios (IRR) with 95% confidence intervals (CI) and p-values were calculated. Testing was carried out with a p-value <0.05 deemed significant.

For peer review only - http://bmjopen.bmj.com/site/about/guidelines.xhtml

RESULTS

Descriptive findings

A full breakdown of the number of licence applications, and percentage issued, in each CIZ and the control group by financial year is provided in the supplementary material (Supplementary Table 1).

From 1 April 2006 to 31 March 2017, 1254 license applications were received by Southwark Council (Table 1). The most common application type was for eateries, with 427 (34.1%) applications made over the 10 years. The number of applications for drinking establishments, off-sales and other outlets were similar; 237 (18.9%), 248 (19.8%) and 255 (20.3%) respectively. During this time period, the council received only 87 (6.9%) applications for takeaways.

Across the period and across types of venue, 38.4% of applications were made within CIZ areas. “Other outlets” (venue type) had the highest proportion of applications within CIZs (47.1%), of which over 80% were made within Bankside CIZ alone. “Off-sales” venue type had the lowest proportion of applications within CIZ areas with 72.6% relating to the control region. Over a quarter (26.4%) of all CIZ applications were made in Bankside. Peckham and Camberwell consistently received fewer applications than Bankside across the period, and for all outlet categories.

Between 2009/10 and 2011/12, following CIZ establishment, the number of issued licenses for drinking establishments, eateries and other outlets decreases (Figure 1). Numbers for off-sales and takeaways remained relatively stable. Although the number of new drinking establishments remained low, there was a general trend upwards in the number of eateries and other outlets between 2011/12 and 2016/17.

Following CIZ implementation the mean number of applications and issued licenses for drinking establishments decreased across the three intervention areas (Table 2). The reductions were not uniform and ranged from a 36% reduction in Peckham to a 100% reduction in Camberwell. Applications and issued licenses for eateries and takeaways increased across the intervention regions. Notably, Peckham experienced a seven fold increase in the mean number of newly issued licenses for eateries and Camberwell experienced a 50% increase. For off-sales, there was a decrease in the number of new licenses issued in both Bankside and Peckham, with Camberwell experiencing a 163% increase. Other outlets increased by 71% in Peckham, with

For peer review only - http://bmjopen.bmj.com/site/about/guidelines.xhtml

the other two intervention areas seeing a decrease in both the mean number of applications and issued licenses.

Regression analysis

Multivariate regression analysis indicated that CIZ establishment (adjusted for overall time trends across all regions) had no discernible effect on the number of applications and issued licenses for drinking establishments across all CIZs and for each CIZ region individually (Table 2). For eateries, CIZ implementation was associated with a 58% increase in the number of licenses issued (IRR = 1.58, 95% CI: 1.02 – 2.52, P = 0.04). This effect appears to be driven by a 937% increase in the number of new eateries in Peckham (IRR = 10.38, 95% CI: 1.39 – 77.66, P = 0.02). There was a statistically significant increase in the number of applications and issued licenses for takeaways across the three intervention regions, adjusted for overall time trends across all regions (IRR = 3.57, 95% CI: 1.23 – 10.24, P = 0.02) and (IRR = 3.89, 95% CI: 1.32 – 11.49, P = 0.01) respectively. On disaggregating the areas, only the number of newly issued takeaways in Bankside achieved significance (IRR = 4.38, 95% CI: 1.20 – 15.91, P = 0.03). For off-sales and other outlets, CIZ establishment had no statistically significant effect on the number of applications and issued licenses across all CIZs. Camberwell CIZ experienced a statistically significant increase in the number of off-sale applications (IRR = 3.65, 95% CI: 1.00 – 13.30, P = 0.05), but the number of issued licenses did not achieve significance. Bankside CIZ experiences a statistically significant decrease in the number of applications for other outlets (IRR = 0.56, 95% CI: 0.33 – 0.95), P = 0.03). Again, no significant effect was found for the number of licenses issued (adjusted for overall time trends across all regions).

For peer review only

Table 1: Number of licence applications made within each CIZ and the control group, by outlet type with percentages indicating proportion of applications by licence type overall.

	Drinking Establishments		Eateries		Takeaways		Off-sales		Other Outlets		TOTAL	
	n	%	n	%	n	%	n	%	n	%	n	%
**All CIZs**	**89**	**37.6**	**172**	**40.3**	**32**	**36.8**	**68**	**27.4**	**120**	**47.1**	**481**	**38.4**
Bankside	*63*		*114*		*18*		*39*		*97*		***331***	
Peckham	*16*		*34*		*9*		*18*		*18*		***95***	
Camberwell	*10*		*24*		*5*		*11*		*5*		***55***	
**Control**	**148**	**62.4**	**255**	**59.7**	**55**	**63.2**	**180**	**72.6**	**135**	**52.9**	**773**	**61.6**
**TOTAL [%]**	**237**	**[18.9]**	**427**	**[34.1]**	**87**	**[6.9]**	**248**	**[19.8]**	**255**	**[20.3]**	**1254**	**[100]**

For peer review only

Table 2: Before and after analysis of Southwark's three CIZs by outlet type, 2006/07 – 2015/16

LICENSE APPLICATIONS AND ISSUED LICENSES BY VENUE TYPE						
				Adjusted model [a]		
	**Pre-CIZ** Mean per financial year	**Post-CIZ** Mean per financial year	**Change**	**IRR**	**(95%CI)**	**p-value**
DRINKING ESTABLISHMENTS						
**Number of applications**						
**All CIZs**	11.5	4.7	-59%	1.433	(0.837 – 2.455)	0.19
Bankside	7.3	4.3	-41%	1.340	(0.745 – 2.411)	0.33
Peckham	2.3	1.5	-36%	1.704	(0.601 – 4.834)	0.32
Camberwell	5.0	0	-100%	-	-	-
**Control**	13.5					
**Number of issued licences**						
**All CIZs**	10.3	4.3	-58%	1.344	(0.763 – 2.368)	0.31
Bankside	7.0	3.8	-46%	1.253	(0.675 – 2.328)	0.47
Peckham	2.3	1.3	-43%	1.556	(0.530 – 4.569)	0.42
Camberwell	3.0	0	-100%	-	-	-
**Control**	8.1					
EATERIES						
**Number of applications**						
**All CIZs**	10.8	12.6	17%	1.484	(0.972 – 2.266)	0.07
Bankside	8.8	9.9	13%	1.223	(0.767 – 1.951)	0.40
Peckham	1.0	3.6	256%	5.083	(1.185 – 21.803)	0.03*
Camberwell	2.0	3.0	50%	1.869	(0.712 – 4.905)	0.20
**Control**	23.2					
**Number of issued licences**						
**All CIZs**	10.0	12.1	21%	1.582	(1.020 – 2.452)	0.04*
Bankside	8.3	9.3	12%	1.260	(0.777 – 2.041)	0.35
Peckham	0.5	3.4	589%	10.377	(1.386 – 77.664)	0.02*
Camberwell	2.0	3.0	50%	1.916	(0.727 – 5.043)	0.18
**Control**	21.1					
TAKEAWAYS						
**Number of applications**						
**All CIZs**	2.0	2.1	7%	3.571	(1.225 – 10.242)	0.02*
Bankside	2.0	2.8	40%	3.500	(0.981 – 12.492)	0.05
Peckham	1.0	1.6	60%	2.400	(0.248 – 23.236)	0.45
Camberwell	1.0	1.3	33%	6.330	(0.660 – 63.639)	0.12

For peer review only - http://bmjopen.bmj.com/site/about/guidelines.xhtml

For peer review only

**Control**	5.0					
**Number of issued licences**						
**All CIZs**	2.0	2.0	0%	3.894	(1.320 – 11.492)	0.01*
Bankside	2.0	2.8	40%	4.375	(1.203 – 15.911)	0.03*
Peckham	1.0	1.2	20%	1.667	(0.161 – 17.257)	0.67
Camberwell	1.0	1.3	33%	7.600	(0.746 – 77.431)	0.09
**Control**	4.5					
OFF-SALES						
**Number of applications**						
**All CIZs**	8.3	5.4	-36%	1.393	(0.773 – 2.510)	0.27
Bankside	5.0	3.0	-40%	1.092	(0.536 – 2.221)	0.81
Peckham	3.0	1.7	-43%	1.275	(0.453 – 3.584)	0.64
Camberwell	1.3	3.5	163%	3.650	(1.002 – 13.298)	0.05*
**Control**	16.4					
**Number of issued licences**						
**All CIZs**	7.3	4.9	-34%	1.535	(0.824 – 2.861)	0.18
Bankside	4.3	3.0	-31%	1.316	(0.625 – 2.769)	0.47
Peckham	2.5	1.1	-54%	1.067	(0.331 – 3.434)	0.91
Camberwell	1.3	3.5	163%	3.350	(0.918 – 12.223)	0.07
**Control**	14.6					
OTHER OUTLETS						
**Number of applications**						
**All CIZs**	10.0	6.9	-31%	0.623	(0.371 – 1.044)	0.07
Bankside	9.3	7.5	-19%	0.556	(0.325 – 0.951)	0.03*
Peckham	1.0	2.3	129%	2.171	(0.466 – 10.121)	0.32
Camberwell	1.0	1.0	0%	1.143	(0.120 – 10.876)	0.91
**Control**	12.3					
**Number of issued licences**						
**All CIZs**	9.0	5.9	-35%	0.601	(0.346 – 1.047)	0.07
Bankside	8.3	6.5	-21%	0.571	(0.321 – 1.013)	0.55
Peckham	1.0	1.7	71%	1.643	(0.339 – 7.962)	0.54
Camberwell	1.0	0.8	-25%	1.041	(0.101 – 10.692)	0.97
**Control**	10.5					

No intervention was implemented in the control area and therefore a mean across all financial years is displayed
**Denotes statistical significant results at the 5% level*
- Too few applications were made to perform regression analysis

a. Licence applications received and issued per intervention areas and per outlet type using Poisson regression; the number of applications per financial year and per area were included as adjustment variables. The number of applications and issued licensed pre-intervention was used as the reference group (IRR = 1.00).

For peer review only - http://bmjopen.bmj.com/site/about/guidelines.xhtml

DISCUSSION

Statement of principal findings

Across the three intervention areas, absolute numbers of issued licenses for drinking establishments decreased and remained low in frequency following CIZ establishment. In Camberwell not a single application for a new drinking establishment was made post intervention. Conversely, the number of new eateries increased year-on-year. The average number of issued licenses for drinking establishments, off-sales and other outlets decreased following CIZ establishment, except for off-sales in Camberwell and other outlets in Peckham. The mean number of eateries and takeaways increased in all areas tested. Regression analyses of outlet type and intervention showed a significant positive association for eateries across all CIZs and for eateries in Peckham CIZ, compared to the control area. For takeaways there was a significant increase in the number of issued licenses both across all CIZs and for Bankside CIZ, compared to the control area.

Strengths and weaknesses in relation to other studies and policy implications

Revised Guidance issued under section 182 of the Licensing Act 2003 states that an application within a CIZ would normally be refused, or subject to limitations, unless the applicant can demonstrate there will be no cumulative impact on the licensing objectives. [12] A common perception therefore follows that CIZs limit alcohol availability in areas already saturated. A previous study in this local authority found no evidence for this effect. [11] Across the five outlet type categories tested, CIZ establishment did not lead to a significant reduction in any particular outlet type, relative to the control area.

The findings from this study support those from previous research that CIZs can be used as a tool to shape local alcohol environments [16], yet this is the first quantitative evidence to substantiate the theories. This effect is best exemplified by Peckham CIZ which was established to tackle late-night anti-social behaviour occurring due to increasing numbers of drinking establishments in the local area. Food-led establishments were exempted from the CIZ policy in Peckham. Following CIZ establishment the area has experienced a significant and substantial increase in licensed eateries.

Statements of Licensing Policy are an opportunity for LAs to set the licensing goals of urban areas *a priori*. Excluding eateries from the CIZ policy in Peckham was successful in drawing new food-led establishments to that area. Although not explicitly stated in Southwark's SoLP, Camberwell CIZ was established to tackle issues relating to street drinking. Yet off-licences in

For peer review only - http://bmjopen.bmj.com/site/about/guidelines.xhtml

Camberwell appeared to increase following CIZ establishment and there were no new applications for drinking establishments in Camberwell post intervention. In Bankside CIZ a non-significant reduction in Drinking Establishments was experienced in the area. Although, this is in line with the goals of the Bankside policy, a significant increase in licensed Takeaways is unlikely to be. These findings imply that descriptive analysis of the absolute numbers of newly licensed premises in a particular geographic location is inadequate. A robust statistical test, which compares intervention areas to a control region, is required to fully appreciate the impact of CIZ policy. The results from this study can be used to inform the renewal of Southwark's SoLP and provide an evidence-base from which licensing goals to diversify the night-time economy can be developed.

Strengths, weaknesses and further work

Stratifying the dataset by financial year, geographic area and outlet type rendered some strata with very few, or in some cases zero, counts. This was particularly an issue for Peckham CIZ and Camberwell CIZ, which accounted for only 7.6% and 4.4% of the data respectively. Statistical power was therefore constrained resulting in an increased risk of type two error.

Aggregation of outlet type into five discrete categories was a subjective task. Although the approach taken was as systematic as possible, and a sample of venues was sense checked against their online descriptions, there remains the possibility for interpretation. For example: an outlet which operates as a café in the daytime, and a bar at night could be categorised as either 'drinking establishment', 'eatery' or even 'other outlet'. To ensure reproducibility of the results, two researchers completed the categorisation process independently, before a final list was agreed.

Limiting outlet type to only five categories was restrictive and may have reduced precision, but necessary to provide suitable observations in each category. Categorising small drinking establishments that, for example offer food, provide seating areas for their customers and close at 23:00, as equal to a 1000-person capacity nightclub with vertical drinking until 6am is potentially problematic. Policy makers may unnecessarily restrict the former type of venue as a consequence. To better identify the types of venues that cause the most harm, further work should consider other methods of stratifying outlet type, for example by opening time and venue capacity.

A number of gaps exist in the literature preventing policy makers from making informed recommendations relating to alcohol-availability interventions. Evidence for alcohol-availability

For peer review only - http://bmjopen.bmj.com/site/about/guidelines.xhtml

polices are from aggregate studies. Given the heterogeneous nature of the outlet types within on- and off-licence categories, it has been recommended that aggregate studies are discontinued. [9] This study provides a quantitative evaluation of CIZs within a major metropolitan area that disaggregates outlet type into five categories. This novel approach exemplifies how local policy can be used to support certain licensing goals and can facilitate the diversification of the night-time economy.

This research provides deeper insight as to the nature of alcohol exposure in different locations within an urban centre. Further work is required to understand which outlet types are associated with the greatest amount of alcohol-related harm.

Southwark is an inner London Borough with a thriving night-time economy. We argue these findings from this research are generalizable to similar urban centres both in the UK and internationally. The methodology employed is reproducible and scalable. Further research should attempt to replicate similar results in other LAs. In order to achieve higher statistical power, similar studies across a cluster of LA, or on a regional or national scale are recommended.

Conclusions

Cumulative Impact Zones can be used as policy levers to shape local alcohol environments to support the licensing goals of specific geographical areas and diversify the night-time economy. While we found no evidence that CIZs are effective at limiting the density of different types of licensed venues, CIZs can be used as a means to encourage certain types of outlet over others and thereby change the tone of an area's night time economy. To further support the development of local licensing policies, further work is required to understand the impact of different outlet types on alcohol-related harm outcomes.

For peer review only

For peer review only - http://bmjopen.bmj.com/site/about/guidelines.xhtml

Figure Legends

Figure 1: Number of issued licences per financial year in CIZ areas, by outlet type.
*Date of CIZ establishment, Peckham and Camberwell.
**Date of CIZ establishment, Bankside.

Acknowledgements

The authors would like to acknowledge Southwark Council's Licensing Authority for providing access to the data for this study and for providing valuable context surrounding the local licensing policy.

Funding

This work was supported by the National Institute for Health Research (NIHR) under the Collaborations for Leadership in Applied Health Research and Care (CLAHRC) programme for North West London. The views expressed in this publication are those of the author(s) and not necessarily those of the NHS, the NIHR or the Department of Health.

Conflict of Interest

None.

Contributors

CS completed the literature review for this study, led on the data analysis and drafted the manuscript. DF provided context as to the local licensing policy. HW supported the development of the statistical model. RP critically reviewed the manuscript and revised a second draft. HW, AP and RP inputted into the data interpretation and revised the final manuscript. All authors have approved the final manuscript.

Data Sharing Statement

No additional data are available.

For peer review only

For peer review only - http://bmjopen.bmj.com/site/about/guidelines.xhtml

Reference List

[1] Babor TF, Babor T, Caetano R, Casswell S, Edwards G, Giesbrecht N. Alcohol: no ordinary commodity: research and public policy. Oxford University Press; 2010.

[2] NHS Digital, Health Survey for England, 2016. [Available from: www.digital.nhs.uk], 2017.

[3] Burton R, Henn C, Lavoie D, O'Connor R, Perkins C, Sweeney K, Greaves F, Ferguson B, Beynon C, Belloni A, Musto V. The public health burden of alcohol and the effectiveness and cost-effectiveness of alcohol control policies: an evidence review. The public health burden of alcohol and the effectiveness and cost-effectiveness of alcohol control policies: an evidence review. 2016

[4] Campbell CA, Hahn RA, Elder R, Brewer R, Chattopadhyay S, Fielding J, Naimi TS, Toomey T, Lawrence B, Middleton JC, Task Force on Community Preventive Services. The effectiveness of limiting alcohol outlet density as a means of reducing excessive alcohol consumption and alcohol-related harms. American journal of preventive medicine. 2009 ;37(6):556-69.

[5] Jackson R, Johnson M, Campbell F, Messina J, Guillaume L, Meier PS, Goyder E, Chilcott J, Payne N. Interventions on control of alcohol price, promotion and availability for prevention of alcohol use disorders in adults and young people. London: The University of Sheffield, for NICE Centre for Public Health Excellence. 2010

[6] Stockwell T, Gruenewald PJ. Controls on the physical availability of alcohol. The essential handbook of treatment and prevention of alcohol problems. 2004 Feb 6:213-33.

[7] Institute of Alcohol Studies. Alcohol consumption factsheet 2013. [Accessed 09/08/2016]. Available from: http://www.ias.org.uk/.

[8] Richardson EA, Shortt NK, Pearce J, Mitchell R. Alcohol-related illness and deaths in Scottish neighbourhoods: is there a relationship with the number of alcohol outlets. Edinburgh: Centre for Research on Environment, Society and Health and Alcohol Focus Scotland. 2014 Oct 2.

[9] Gmel G, Holmes J, Studer J. Are alcohol outlet densities strongly associated with alcohol-related outcomes? A critical review of recent evidence. Drug and alcohol review. 2016 Jan;35(1):40-54.

[10] Licensing Act 2003. England and Wales. London: HMSO 2003

[11] Sharpe CA, Poots AJ, Watt H, Franklin D, Pinder RJ. Controlling alcohol availability through local policy: an observational study to evaluate Cumulative Impact Zones in a London borough. Journal of Public Health. 2017 Dec 11.

[12] Home Office. Revised Guidance issued under section 182 of the Licensing Act 2003. London: HMSO 2015

For peer review only - http://bmjopen.bmj.com/site/about/guidelines.xhtml

[13] Home Office. Alcohol and late night refreshment licensing England and Wales. National Statistics 2016.

[14] Humphreys DK, Smith DM. Alcohol licensing data: why is it an underused resource in public health?. Health & place. 2013 Nov 1;24:110-4.

[15] Grace D, Egan M, Lock K. Examining local processes when applying a cumulative impact policy to address harms of alcohol outlet density. Health & place. 2016 Jul 1;40:76-82.

[16] Egan M, Brennan A, Buykx P, De Vocht F, Gavens L, Grace D, Halliday E, Hickman M, Holt V, Mooney JD, Lock K. Local policies to tackle a national problem: Comparative qualitative case studies of an English local authority alcohol availability intervention. Health & place. 2016 Sep 1;41:11-8.

[17] Greater London Authority, Culture and the night-time economy. London, GLA, 2017.

[18] Civica. Civica Community Portection Overview 2016. [Accessed 23/07/2016]. Available from: https://www.civica.co.uk/public-protection/overview.

[19] Pitney Bowes. Data Sheet: MapInfo Location Intelligence [Available from: www.mapinfo.com Accessed 21/07/2016.

[20] Bland M. An introduction to medical statistics. Oxford University Press (UK); 2015.

[21] “Stata Statistical Software [program].,” vol. 13 version. College Station: TX:, 13 version. College Station: TX:.

For peer review only

For peer review only - http://bmjopen.bmj.com/site/about/guidelines.xhtml

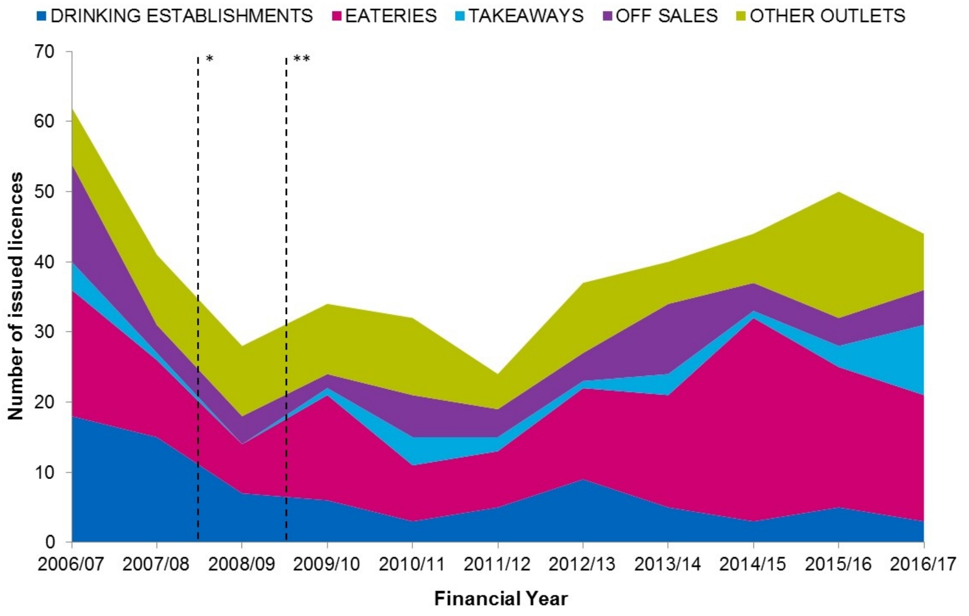

Figure 1: Number of issued licences per financial year in CIZ areas, by outlet type.
*Date of CIZ establishment, Peckham and Camberwell.
**Date of CIZ establishment, Bankside.

199x127mm (150 x 150 DPI)

Supplementary Material

Supplementary Table 1: Number of licence applications and percentage issued in each CIZ and the control group by financial year. Data are stratified by outlet type (five categories).

	2006/ 07	2007/ 08	2008/ 09	2009/ 10	2010/ 11	2011/ 12	2012/ 13	2013/ 14	2014/ 15	2015/ 16	2016/ 17	Total
**DRINKING ESTABLISHMENTS**												
**All CIZs**	**21**	**17**	**7**	**6**	**4**	**5**	**11**	**5**	**3**	**5**	**5**	**89**
% Issued	85.7	88.2	100	100	75.0	100	81.8	100	100	100	60.0	88.8
Bankside	**11**	**11**	**5**	**4**	**4**	**5**	**10**	**3**	**2**	**5**	**3**	**63**
% Issued	100	90.9	100	100	75.0	100	80.0	100	100	100	66.7	92.1
Peckham	**3**	**3**	**2**	**2**	**0**	**0**	**1**	**2**	**1**	**0**	**2**	**16**
% Issued	100	100	100	100	-	-	100	100	100	-	50.0	93.8
Camberwell	**7**	**3**	**0**	**0**	**0**	**0**	**0**	**0**	**0**	**0**	**0**	**10**
% Issued	57.1	66.7	-	-	-	-	-	-	-	-	-	60.0
**Control**	**47**	**19**	**11**	**3**	**6**	**8**	**6**	**6**	**15**	**7**	**20**	**148**
% Issued	91.5	73.7	81.8	66.7	83.3	75.0	100	83.3	80.0	85.7	85.0	84.5
**EATERIES**												
**All CIZs**	**18**	**12**	**9**	**15**	**8**	**8**	**14**	**18**	**29**	**21**	**20**	**172**
% Issued	100	91.7	77.8	100	100	100	92.9	88.9	100	95.2	90.0	94.8
Bankside	**15**	**9**	**6**	**10**	**4**	**6**	**9**	**9**	**17**	**16**	**13**	**114**
% Issued	100	88.9	83.3	100	100	100	88.9	88.9	100	93.8	84.6	93.9
Peckham	**1**	**0**	**2**	**3**	**2**	**1**	**5**	**5**	**7**	**5**	**3**	**34**
% Issued	100	0.0	50.0	100	100	100	100	80.0	100	100	100.0	94.1
Camberwell	**2**	**3**	**1**	**2**	**2**	**1**	**0**	**4**	**5**	**0**	**4**	**24**
% Issued	100	100	100	100	100	100	-	100	100	-	100	100
**Control**	**49**	**17**	**15**	**14**	**18**	**19**	**13**	**21**	**19**	**31**	**39**	**255**
% Issued	93.9	94.1	93.3	85.7	94.4	100	100	85.7	84.2	77.4	94.9	91.0
**TAKEAWAYS**												
**All CIZs**	**4**	**1**	**0**	**2**	**4**	**2**	**1**	**3**	**1**	**3**	**11**	**32**
% Issued	100	100	-	50.0	100	100	100	100	100	100	90.9	93.8
Bankside	**3**	**0**	**0**	**1**	**3**	**2**	**1**	**0**	**0**	**2**	**6**	**18**
% Issued	100	-	-	100	100	100	100	-	-	100	100	100
Peckham	**0**	**1**	**0**	**1**	**0**	**0**	**0**	**1**	**1**	**1**	**4**	**9**
% Issued	-	100	-	0	-	-	-	100	100	100	75.0	77.8
Camberwell	**1**	**0**	**0**	**0**	**1**	**0**	**0**	**2**	**0**	**0**	**1**	**5**
% Issued	100	-	-	-	100	-	-	100	-	-	100	100
**Control**	**19**	**6**	**2**	**1**	**1**	**6**	**1**	**4**	**3**	**5**	**7**	**55**
% Issued	100	83.3	100	100	100	66.7	100	100	100	100	71.4	90.9
**OFF-SALES**												
**All CIZs**	**14**	**5**	**6**	**2**	**7**	**6**	**4**	**11**	**4**	**4**	**5**	**68**
% Issued	100	80.0	66.7	100	85.7	66.7	100	90.9	100	100	100	89.7
Bankside	**7**	**3**	**5**	**1**	**5**	**1**	**1**	**5**	**4**	**3**	**4**	**39**

For peer review only

% Issued	100	100	60.0	100	100	100	100	100	100	100	100	94.9
Peckham	**5**	**1**	**0**	**1**	**2**	**2**	**3**	**2**	**0**	**1**	**1**	**18**
% Issued	100.0	-	-	100	50.0	-	100	50.0	-	100	100	72.2
Camberwell	**2**	**1**	**1**	**0**	**0**	**3**	**0**	**4**	**0**	**0**	**0**	**11**
% Issued	100	100	100	-	-	100	-	100	-	-	-	100
**Control**	**40**	**18**	**15**	**11**	**9**	**17**	**10**	**18**	**16**	**16**	**10**	**180**
% Issued	92.5	94.4	86.7	81.8	100	100	100	100	81.3	68.8	70.0	89.4
**OTHER OUTLETS**												
**All CIZs**	**9**	**10**	**12**	**12**	**12**	**6**	**10**	**9**	**8**	**21**	**11**	**120**
% Issued	88.9	100	83.3	83.3	91.7	83.3	100	66.7	87.5	85.7	72.7	85.8
Bankside	**7**	**10**	**10**	**12**	**9**	**5**	**7**	**6**	**7**	**15**	**9**	**97**
% Issued	85.7	100	90.0	83.3	100	80.0	100	66.7	100	80.0	77.8	87.6
Peckham	**1**	**0**	**2**	**0**	**2**	**1**	**3**	**3**	**0**	**5**	**1**	**18**
% Issued	100	-	50.0	0.0	50.0	100	100	66.7	-	100	0.0	77.8
Camberwell	**1**	**0**	**0**	**0**	**1**	**0**	**0**	**0**	**1**	**1**	**1**	**5**
% Issued	100	-	-	-	100	-	-	-	0.0	100	100	80.0
**Control**	**18**	**6**	**5**	**12**	**8**	**10**	**10**	**11**	**19**	**20**	**16**	**135**
% Issued	94.4	100	60.0	83.3	100	100	100	100	78.9	80.0	62.5	85.9

For peer review only

STROBE 2007 (v4) checklist of items to be included in reports of observational studies in epidemiology*

Checklist for cohort, case-control, and cross-sectional studies (combined)

**Section/Topic**	**Item #**	**Recommendation**	**Reported on page**
**Title and abstract**	1	(*a*) Indicate the study's design with a commonly used term in the title or the abstract	1
		(*b*) Provide in the abstract an informative and balanced summary of what was done and what was found	2
**Introduction**			
Background/rationale	2	Explain the scientific background and rationale for the investigation being reported	4-6
Objectives	3	State specific objectives, including any pre-specified hypotheses	5
**Methods**			
Study design	4	Present key elements of study design early in the paper	2
Setting	5	Describe the setting, locations, and relevant dates, including periods of recruitment, exposure, follow-up, and data collection	2 and 7
Participants	6	(*a*) *Cohort study*—Give the eligibility criteria, and the sources and methods of selection of participants. Describe methods of follow-up<br>*Case-control study*—Give the eligibility criteria, and the sources and methods of case ascertainment and control selection. Give the rationale for the choice of cases and controls<br>*Cross-sectional study*—Give the eligibility criteria, and the sources and methods of selection of participants	Data source is explained on page (this is data that wa readily available)
		(*b*) *Cohort study*—For matched studies, give matching criteria and number of exposed and unexposed<br>*Case-control study*—For matched studies, give matching criteria and the number of controls per case	N/A
Variables	7	Clearly define all outcomes, exposures, predictors, potential confounders, and effect modifiers. Give diagnostic criteria, if applicable	7
Data sources/ measurement	8*	For each variable of interest, give sources of data and details of methods of assessment (measurement). Describe comparability of assessment methods if there is more than one group	7
Bias	9	Describe any efforts to address potential sources of bias	7 (two adjustmen variables were included in the statistical model)
Study size	10	Explain how the study size was arrived at	7 (all licence applications within defined time perio were included, a study sample was therefore not

For peer review only

			necessary)
Quantitative variables	11	Explain how quantitative variables were handled in the analyses. If applicable, describe which groupings were chosen and why	7
Statistical methods	12	(*a*) Describe all statistical methods, including those used to control for confounding	7
		(*b*) Describe any methods used to examine subgroups and interactions	7
		(*c*) Explain how missing data were addressed	N/A (a comprehensive dat[illegible] set was sourced)
		(*d*) *Cohort study*—If applicable, explain how loss to follow-up was addressed<br>*Case-control study*—If applicable, explain how matching of cases and controls was addressed<br>*Cross-sectional study*—If applicable, describe analytical methods taking account of sampling strategy	N/A
		(*e*) Describe any sensitivity analyses	N/A
**Results**			
Participants	13*	(a) Report numbers of individuals at each stage of study—eg numbers potentially eligible, examined for eligibility, confirmed eligible, included in the study, completing follow-up, and analysed	N/A
		(b) Give reasons for non-participation at each stage	N/A
		(c) Consider use of a flow diagram	N/A
Descriptive data	14*	(a) Give characteristics of study participants (eg demographic, clinical, social) and information on exposures and potential confounders	N/A
		(b) Indicate number of participants with missing data for each variable of interest	7
		(c) *Cohort study*—Summarise follow-up time (eg, average and total amount)	N/A
Outcome data	15*	*Cohort study*—Report numbers of outcome events or summary measures over time	10, 11-12 (see als[illegible] supplementary material)
		Case-control study—Report numbers in each exposure category, or summary measures of exposure	10, 11-12 (see als[illegible] supplementary material)
		Cross-sectional study—Report numbers of outcome events or summary measures	10, 11-12 (see als[illegible] supplementary material)
Main results	16	(*a*) Give unadjusted estimates and, if applicable, confounder-adjusted estimates and their precision (eg, 95% confidence interval). Make clear which confounders were adjusted for and why they were included	11-12
		(*b*) Report category boundaries when continuous variables were categorized	N/A
		(*c*) If relevant, consider translating estimates of relative risk into absolute risk for a meaningful time period	N/A

For peer review only

Other analyses	17	Report other analyses done—eg analyses of subgroups and interactions, and sensitivity analyses	10, 11-12
**Discussion**			
Key results	18	Summarise key results with reference to study objectives	13
Limitations	19	Discuss limitations of the study, taking into account sources of potential bias or imprecision. Discuss both direction and magnitude of any potential bias	13-14
Interpretation	20	Give a cautious overall interpretation of results considering objectives, limitations, multiplicity of analyses, results from similar studies, and other relevant evidence	13-15
Generalisability	21	Discuss the generalisability (external validity) of the study results	15
**Other information**			
Funding	22	Give the source of funding and the role of the funders for the present study and, if applicable, for the original study on which the present article is based	16

*Give information separately for cases and controls in case-control studies and, if applicable, for exposed and unexposed groups in cohort and cross-sectional studies.

Note: An Explanation and Elaboration article discusses each checklist item and gives methodological background and published examples of transparent reporting. The STROBE checklist is best used in conjunction with this article (freely available on the Web sites of PLoS Medicine at http://www.plosmedicine.org/, Annals of Internal Medicine at http://www.annals.org/, and Epidemiology at http://www.epidem.com/). Information on the STROBE Initiative is available at www.strobe-statement.org.

For peer review only

BMJ Open

An observational study to examine how Cumulative Impact Zones influence alcohol availability from different types of licensed outlets in an inner London Borough

Journal:	*BMJ Open*
Manuscript ID	bmjopen-2018-027320.R1
Article Type:	Original research
Date Submitted by the Author:	15-Mar-2019
Complete List of Authors:	Sharpe, Carolyn; Imperial College London School of Public Health, Department of Primary Care and Public Health; Southwark Council, Public Health Directorate<br>Poots, Alan; Imperial College London, NIHR CLARHC Northwest London; Independent Researcher<br>Watt, Hilary; Imperial College London, Primary Care and Public Health<br>Williamson, Chris; Southwark Council, Public Health Division, Place & Wellbeing Department<br>Franklin, David; Southwark Council, Licensing<br>Pinder, Richard; Imperial College London School of Public Health, Department of Primary Care and Public Health; Southwark Council, Public Health Directorate
**Primary Subject Heading**:	Public health
Secondary Subject Heading:	Health policy
Keywords:	PUBLIC HEALTH, Cumulative Impact Zone, Alcohol Licensing, Alcohol Availability

SCHOLARONE™
Manuscripts

An observational study to examine how Cumulative Impact Zones influence alcohol availability from different types of licensed outlets in an inner London Borough

Authors:

C A Sharpe, Policy Officer, Imperial College London & Southwark Council[1,2]
carolyn.sharpe1@nhs.net – *corresponding & first author*

Dr A J Poots, Principal Information Analyst, formerly Imperial College London[4,a]

H Watt, Consultant Statistician & Teaching Fellow, Imperial College London[1]

Chris Williamson, Head of Public Health Intelligence, Southwark Council[2]

D Franklin, Head of Licensing, Southwark Council[3]

Dr R J Pinder, Consultant in Public Health, Imperial College London & Southwark Council[1,2]

Affiliations at the time of study:

1. Department of Primary Care and Public Health, School of Public Health, Imperial College London, Reynolds Building, St Dunstans Road, London, W6 8RP, United Kingdom

2. Public Health Directorate, Southwark Council, 1st Floor, Hub 1, PO Box 64529, London, SE1P 5LX, United Kingdom

3. Licensing Team, Southwark Council, 3rd Floor, Hub 1, PO Box 64529, London, SE1P 5LX, United Kingdom

4. NIHR CLARHC Northwest London, Imperial College London, Floor 4, Lift Bank D, Chelsea and Westminster Hospital, 369 Fulham Road, London, SW10 9NH, United Kingdom

Current affiliations:

a) Independent researcher

Word Count: 3168 words
(4000 word limit)

For peer review only - http://bmjopen.bmj.com/site/about/guidelines.xhtml

For peer review only

Abstract (298 / 300 words)

OBJECTIVES: Cumulative Impact Zones (CIZs) are a widely implemented local policy intended to restrict alcohol availability in areas proliferated with licensed outlets. Limited previous research has questioned their effectiveness and suggested they may play a more nuanced role in shaping local alcohol environments. This study evaluates the association between CIZ implementation and the number of licence applications made and issued for five outlet categories, relative to a control region.

DESIGN: A quantitative observational study.

SETTING: The London Borough of Southwark, an inner London borough, which currently enforces three CIZs.

POPULATION: Licence applications received by Southwark Council's Licensing Authority between 1 April 2006 and 31 March 2017 (N = 1254).

INTERVENTIONS: CIZ implementation.

PRIMARY OUTCOME MEASURES: Five outlet types were categorised and evaluated: Drinking Establishments, Eateries, Takeaways, Off Sales, and Other Outlets. Primary outcome measures were the number of applications received and the number of licences issued. These were analysed using Poisson regression of counts over time.

RESULTS: Across all CIZs, implementation was associated with an increase in the number of eateries (IRR = 1.58, 95% CI: 1.02 – 2.52, P = 0.04) and takeaways (IRR = 3.89, 95% CI: 1.32 – 11.49, P = 0.01), compared to the control area, adjusting for time trends. No discernible association was found for the remaining outlet types. Disaggregating by area indicated a 10-fold increase in the number of new eateries in Peckham CIZ (IRR = 10.38, 95% CI: 1.39 – 77.66, P = 0.02) and a four-fold increase in the number of newly licensed takeaways in Bankside CIZ (IRR = 4.38, 95% CI: 1.20 – 15.91, P = 0.03), compared to the control area.

CONCLUSIONS: Cumulative Impact Zones may be useful as policy levers to shape local alcohol environments to support the licensing goals of specific geographical areas and diversify the night-time economy.

For peer review only

Article Summary

Strengths and limitations of this study

- This study provides the first quantitative evaluation of CIZs that disaggregates outlet type into five discrete categories.
- This research provides deeper insight in to the nature of alcohol exposure in different locations within an urban centre and therefore provides an evidence base from which local policy can be developed to support local licensing goals which may include diversification of the night-time economy.
- This study shows that descriptive analyses of alcohol control policies (using absolute numbers) are insufficient, and provides a robust statistical approach that can be employed in other urban settings.
- Although substantial steps were taken to assure the reproducibility of the results, the categorisation of outlet type into five discrete categories involved qualitative assessment and there remains a risk of subjectivity.
- Necessary stratification of the dataset rendered some strata with very few (or in some cases zero) counts, thereby constraining statistical power.

Key words

Public Health, Cumulative Impact Zone, Alcohol Licensing, Alcohol Availability

For peer review only

Main Text

INTRODUCTION

Alcohol harm and opportunities to control availability

Alcohol occupies a prominent role in modern society. It is a commodity used by many, across social strata, as a relaxant and a means of enjoyment [1]. The misuse of alcohol leads to increased morbidity and mortality. In England in 2016, it was estimated that almost 13 million adults drank at levels that increased their risk of harm. [2] Among those aged 15 to 49, alcohol has become the leading risk factor for ill-health, early mortality, and disability. [3]

Outlet spatial density is positively associated with consumption. [4] [5] Policies to restrict the physical availability of alcohol are well supported in the literature. [6] However, such evidence is based on aggregate studies that consider all alcohol-outlet types as equal. At best, outlets have been categorised as either on or off-sales. On-sales of alcohol refer to venues such as pubs, nightclubs and restaurants where alcohol is purchased to be consumed on the premises. Off-sales of alcohol refer to premises such as off-licenses and supermarkets from which alcohol is purchased to be consumed away from the retailer's premises. [7] Although greater association has been found between off-sales and alcohol-related hospitalisations and deaths than with on-sales, [8] both on- and off-licences cover a broad range of establishments. Such aggregate studies remain a crude way to evaluate alcohol availability. [9]

A full description of the licensing process in England and Wales, as defined by the Licensing Act 2003, [10] is outlined in our previous paper. [11] In summary, Revised Guidance issued under section 182 of the Licensing Act 2003 enables local licensing authorities (LAs) to address the cumulative impact of a concentration of licensed premises. [12] Cumulative Impact is defined as "the potential impact on the promotion of the licensing objectives of a significant number of licensed premises concentrated in one area". [12] The licensing objectives, in England and Wales, as defined by the Licensing Act 2003 are: the prevention of crime and disorder; public safety; the prevention of public nuisance; and the protection of children from harm [10].

A CIZ may therefore be established, if a LA can evidentially justify that the licensing objectives are not being upheld in a particular location. This evidence must be published in a LA's Statement of Licensing Policy (SOLP). The Licensing Act 2003 requires all LAs to develop and publish a SOLP at least every five years. [10] Evidence to establish a CIZ relates to the licensing objectives. Local crime, disorder and anti-social behaviour statistics can be evaluated alongside acute health data, such as alcohol-related ambulance

For peer review only - http://bmjopen.bmj.com/site/about/guidelines.xhtml

attendances ad hospital admissions. Environmental health complaints, particularly in relation to litter and noise, can also be included. [12]

Current evidence relating to CIZs

In 2016 there were 215 CIZs established across 106 LAs [13]. Despite their widespread establishment, there is a scarcity of literature evaluating their impact. [14] Revised Guidance issued under section 182 of the Licensing Act 2003 states that an application within a CIZ would normally be refused, or subject to limitations, unless the applicant can demonstrate there will be no cumulative impact on the licensing objectives. [12] A common perception therefore follows that CIZs limit alcohol availability in areas already saturated. [15] Previous work undertaken in the London Borough of Southwark found no evidence that CIZ establishment reduced the number of licence applications or issued licenses across its three policy areas combined, when compared to a control region. On analysing one CIZ area in isolation, establishment was associated with a statistically significant 133% increase in successful applications. [11]

Qualitative research, conducted in 2016, investigating the drivers behind CIZ implementation has suggested them to be more nuanced than simply restricting outlet density. [16] Participants described how CIZs have evolved to encourage local regeneration goals. CIZs tended not to be established to cap the number of licenced outlets, rather they are viewed as a tool to control the temporal availability of alcohol and even to encourage a certain type of application e.g. from arts, food and coffee led establishments as opposed to 'vertical drinking' bars.

Plans to diversify night-time economies and offer alternatives to alcohol-led activities are emerging. In April 2017, the Mayor of London published guidance for culture and the night-time economy in the capital and identified diversification and inclusiveness as key areas of focus. Through encouraging leisure and cultural venues e.g.: late night markets, museums, shops, cafes, theatres, and fairs, urban centres hope to a wider clientele. Examples of which include non-drinkers, older people, families and those with a disability. [17] Despite these plans, an evidence base in support of diversifying the night-time economy is lacking.

Gaps in the literature

A key research gap exists around the assumption that all on- and off-licensed outlets are treated as equal. [9] Although aggregate studies may show an association between outlet density and harm, they are of limited use to policy makers. Interventions need to be targeted towards outlets that cause the most harm. Therefore, through stratifying by outlet type, we aim to understand how CIZ establishment is associated with the number of licence

For peer review only - http://bmjopen.bmj.com/site/about/guidelines.xhtml

applications and issued licences for different outlet types. Qualitative evidence indicates that CIZs can facilitate the proliferation of certain types of premises, thereby diversifying local night-time economies. [16] We therefore propose a quantitative methodology to test for this.

Southwark's three cumulative impact zones

Southwark is an inner London borough with an estimated population of 311,655. [18] Southwark is home to approximately 1,400 licensed premises which make a significant contribution to its culture and economy. However, some locations within the borough have reached saturation point. Within these areas of saturation, Southwark enforces three CIZs (Figure 1); Camberwell CIZ and Peckham CIZ were both established 05 November 2008. Bankside CIZ was established a year later on 05 November 2009. The implementation of each CIZ was to achieve a unique objective. (Franklin D. Head of Licensing, Southwark Council. Personal communication. 17 July 2018) With almost 70% of all CIZ licence applications falling within Bankside alone, the intervention aimed to tackle binge drinking-related issues. The Bankside CIZ policy applies to all venue types except for theatres and cinemas. Peckham and Camberwell CIZs were established to tackle late-night anti-social behaviour and street drinking respectively. Notably, food-led establishments, such as restaurants and cafes, are exempt from both CIZ policies. The size and estimated population of Southwark's CIZ areas is detailed in the supplementary information (Supplementary Table 1).

For peer review only

For peer review only - http://bmjopen.bmj.com/site/about/guidelines.xhtml

METHODS

Data collection and collation

A comprehensive alcohol licensing data set of all applications made within Southwark between 1 April 2006 and 31 March 2017 was obtained from Authority Public Protection (APP); a database used by the council to manage licence applications. [19] Licensing data prior to 2006 could not be analysed by year of application and year of issue, as in 2005 Southwark transitioned their licensing data to APP from a previous data base. Geographical information software (GIS) [20] was used to assign each outlet application an easting and northing. These coordinates were then used to identify applications within each CIZ region and the control group. The issue date was used as a proxy that applications were successful. All applications without an issue date were categorised as having been rejected or withdrawn. An outlet type field was obtained from APP containing 100 different entries for outlet type. This field was aggregated into five mutually-exclusive categories;

- Bars, public-houses, hotel bars and nightclubs were grouped and are collectively referred to as **drinking establishments**
- Restaurants and cafes were aggregated together and are collectively referred to as **eateries**
- Licensed takeaway food restaurants were kept separate and are referred to as **takeaways**
- Off-licences, grocery stores and supermarkets are grouped into one **off-sales** category
- Those alcohol-outlets that do not fit into any of the above categories are collectively referred to as **other outlets** (such as florists, boats and schools).

To ensure reproducibility of the results, two researchers completed the categorisation process independently, before a final list was agreed.

Licence applications were grouped by financial year (April to the following March) starting from 2006/07. An indicator variable for the intervention was fitted, to indicate which time periods in which regions had a CIZ in operation. The three CIZs were established on 5 November in each of the respective years. We therefore grouped applications in 365-day periods relative to the date of CIZ establishment.

Statistical analyses

Poisson regression [21] was employed to model the association between CIZ establishment (indicator variable) and firstly the number of applications and secondly the number of licenses issued. We adjusted for financial yearly trend, across all regions tested, and for geographical area, including both in the model as categorical adjustment variables; financial

For peer review only - http://bmjopen.bmj.com/site/about/guidelines.xhtml

yearly trend did not fit well enough to a linear model for that to be a viable alternative. An adjustment was made to accommodate for the CIZs being established mid-financial year. Statistical analyses were executed using STATA 13.0. [22] Incidence rate ratios (IRR's which represent ratios of counts) with 95% confidence intervals (CI) and p-values were calculated. Testing was carried out with a p-value <0.05 deemed significant.

Patient and Public Involvement

There was no patient or public involvement in this study.

For peer review only

For peer review only - http://bmjopen.bmj.com/site/about/guidelines.xhtml

RESULTS

Descriptive findings

A full breakdown of the number of licence applications, and percentage issued, in each CIZ and the control group by financial year is provided in the supplementary material (Supplementary Table 2).

From 1 April 2006 to 31 March 2017, 1254 license applications were received by Southwark Council (Table 1). The most common application type was for eateries, with 427 (34.1%) applications made over the 10 years. The number of applications for drinking establishments, off-sales and other outlets were similar; 237 (18.9%), 248 (19.8%) and 255 (20.3%) respectively. During this time period, the council received only 87 (6.9%) applications for takeaways.

Across the period and across types of venue, 38.4% of applications were made within CIZ areas. “Other outlets” (venue type) had the highest proportion of applications within CIZs (47.1%), of which over 80% were made within Bankside CIZ alone. “Off-sales” venue type had the lowest proportion of applications within CIZ areas with 72.6% relating to the control region. Over a quarter (26.4%) of all new applications were made in Bankside. Peckham and Camberwell consistently received fewer applications than Bankside across the period, and for all outlet categories.

Between 2009/10 and 2011/12, following CIZ establishment, the number of issued licenses for drinking establishments, eateries and other outlets decreases (Figure 2). Numbers for off-sales and takeaways remained relatively stable. Although the number of new drinking establishments remained low, there was a general trend upwards in the number of new eateries and “other outlets” between 2011/12 and 2016/17.

Following CIZ implementation, the mean number of applications and issued licenses for drinking establishments decreased across the three intervention areas (Table 2). The reductions were not uniform and ranged from a 36% reduction in Peckham to a 100% reduction in Camberwell. Applications and issued licenses for eateries and takeaways increased across the intervention regions. Notably, Peckham experienced a seven-fold increase in the mean number of newly issued licenses for eateries and Camberwell experienced a 50% increase. For off-sales, there was a decrease in the number of new licenses issued in both Bankside and Peckham, with Camberwell experiencing a 163% increase. Other outlets increased by 71% in Peckham, with the other two intervention areas seeing a decrease in both the mean number of applications and issued licenses.

For peer review only - http://bmjopen.bmj.com/site/about/guidelines.xhtml

Regression analysis

Multivariate regression analysis indicated that there was no discernible association between CIZ establishment (adjusted for overall time trends across all regions) and the number of applications and issued licenses for drinking establishments across all CIZs and for each CIZ region individually (Table 2). For eateries, CIZ implementation was associated with a 58% increase in the number of licenses issued (IRR = 1.58, 95% CI: 1.02 – 2.52, P = 0.04). This association appears to be driven by a 937% increase in the number of new eateries in Peckham (IRR = 10.38, 95% CI: 1.39 – 77.66, P = 0.02). There was a statistically significant increase in the number of applications and issued licenses for takeaways across the three intervention regions, adjusted for overall time trends across all regions (IRR = 3.57, 95% CI: 1.23 – 10.24, P = 0.02) and (IRR = 3.89, 95% CI: 1.32 – 11.49, P = 0.01) respectively. On disaggregating the areas, only the number of newly issued takeaways in Bankside achieved significance (IRR = 4.38, 95% CI: 1.20 – 15.91, P = 0.03). For off-sales and other outlets, there was no statistically significant association between CIZ establishment and the number of applications and issued licenses across all CIZs. Camberwell CIZ experienced a statistically significant increase in the number of off-sale applications (IRR = 3.65, 95% CI: 1.00 – 13.30, P = 0.05), but the number of issued licenses did not achieve significance. Bankside CIZ experiences a statistically significant decrease in the number of applications for other outlets (IRR = 0.56, 95% CI: 0.33 – 0.95), P = 0.03). Again, no significant association was found for the number of licenses issued (adjusted for overall time trends across all regions).

For peer review only

For peer review only - http://bmjopen.bmj.com/site/about/guidelines.xhtml

Table 1: Number of licence applications made within each CIZ and the control group, by outlet type with percentages indicating, for each licence type, the proportion in the CIZ areas or the control area.

	Drinking Establishments		Eateries		Takeaways		Off-sales		Other Outlets		TOTAL	
	n	%	n	%	n	%	n	%	n	%	n	%
**All CIZs**	**89**	**37.6**	**172**	**40.3**	**32**	**36.8**	**68**	**27.4**	**120**	**47.1**	**481**	**38.4**
Bankside	*63*		*114*		*18*		*39*		*97*		***331***	
Peckham	*16*		*34*		*9*		*18*		*18*		***95***	
Camberwell	*10*		*24*		*5*		*11*		*5*		***55***	
**Control**	**148**	**62.4**	**255**	**59.7**	**55**	**63.2**	**180**	**72.6**	**135**	**52.9**	**773**	**61.6**
**TOTAL [%]**	**237**	**[18.9]**	**427**	**[34.1]**	**87**	**[6.9]**	**248**	**[19.8]**	**255**	**[20.3]**	**1254**	**[100]**

For peer review only

For peer review only - http://bmjopen.bmj.com/site/about/guidelines.xhtml

Table 2: Before and after analysis of Southwark's three CIZs by outlet type, 2006/07 – 2015/16

LICENSE APPLICATIONS AND ISSUED LICENSES BY VENUE TYPE						
				Adjusted model [a]		
	**Pre-CIZ** Mean per financial year	**Post-CIZ** Mean per financial year	**Change**	**IRR**	**(95%CI)**	**p-value**
DRINKING ESTABLISHMENTS						
**Number of applications**						
**All CIZs**	11.5	4.7	-59%	1.433	(0.837 – 2.455)	0.19
Bankside	7.3	4.3	-41%	1.340	(0.745 – 2.411)	0.33
Peckham	2.3	1.5	-36%	1.704	(0.601 – 4.834)	0.32
Camberwell	5.0	0	-100%	-	-	-
**Control**	13.5					
**Number of issued licences**						
**All CIZs**	10.3	4.3	-58%	1.344	(0.763 – 2.368)	0.31
Bankside	7.0	3.8	-46%	1.253	(0.675 – 2.328)	0.47
Peckham	2.3	1.3	-43%	1.556	(0.530 – 4.569)	0.42
Camberwell	3.0	0	-100%	-	-	-
**Control**	8.1					
EATERIES						
**Number of applications**						
**All CIZs**	10.8	12.6	17%	1.484	(0.972 – 2.266)	0.07
Bankside	8.8	9.9	13%	1.223	(0.767 – 1.951)	0.40
Peckham	1.0	3.6	256%	5.083	(1.185 – 21.803)	0.03*
Camberwell	2.0	3.0	50%	1.869	(0.712 – 4.905)	0.20
**Control**	23.2					
**Number of issued licences**						
**All CIZs**	10.0	12.1	21%	1.582	(1.020 – 2.452)	0.04*
Bankside	8.3	9.3	12%	1.260	(0.777 – 2.041)	0.35
Peckham	0.5	3.4	589%	10.377	(1.386 – 77.664)	0.02*
Camberwell	2.0	3.0	50%	1.916	(0.727 – 5.043)	0.18
**Control**	21.1					
TAKEAWAYS						
**Number of applications**						
**All CIZs**	2.0	2.1	7%	3.571	(1.225 – 10.242)	0.02*
Bankside	2.0	2.8	40%	3.500	(0.981 – 12.492)	0.05
Peckham	1.0	1.6	60%	2.400	(0.248 – 23.236)	0.45
Camberwell	1.0	1.3	33%	6.330	(0.660 – 63.639)	0.12
**Control**	5.0					
**Number of issued licences**						
**All CIZs**	2.0	2.0	0%	3.894	(1.320 – 11.492)	0.01*
Bankside	2.0	2.8	40%	4.375	(1.203 – 15.911)	0.03*

For peer review only - http://bmjopen.bmj.com/site/about/guidelines.xhtml

For peer review only

Peckham	1.0	1.2	20%	1.667	(0.161 – 17.257)	0.67
Camberwell	1.0	1.3	33%	7.600	(0.746 – 77.431)	0.09
**Control**	4.5					
OFF-SALES						
**Number of applications**						
**All CIZs**	8.3	5.4	-36%	1.393	(0.773 – 2.510)	0.27
Bankside	5.0	3.0	-40%	1.092	(0.536 – 2.221)	0.81
Peckham	3.0	1.7	-43%	1.275	(0.453 – 3.584)	0.64
Camberwell	1.3	3.5	163%	3.650	(1.002 – 13.298)	0.05*
**Control**	16.4					
**Number of issued licences**						
**All CIZs**	7.3	4.9	-34%	1.535	(0.824 – 2.861)	0.18
Bankside	4.3	3.0	-31%	1.316	(0.625 – 2.769)	0.47
Peckham	2.5	1.1	-54%	1.067	(0.331 – 3.434)	0.91
Camberwell	1.3	3.5	163%	3.350	(0.918 – 12.223)	0.07
**Control**	14.6					
OTHER OUTLETS						
**Number of applications**						
**All CIZs**	10.0	6.9	-31%	0.623	(0.371 – 1.044)	0.07
Bankside	9.3	7.5	-19%	0.556	(0.325 – 0.951)	0.03*
Peckham	1.0	2.3	129%	2.171	(0.466 – 10.121)	0.32
Camberwell	1.0	1.0	0%	1.143	(0.120 – 10.876)	0.91
**Control**	12.3					
**Number of issued licences**						
**All CIZs**	9.0	5.9	-35%	0.601	(0.346 – 1.047)	0.07
Bankside	8.3	6.5	-21%	0.571	(0.321 – 1.013)	0.55
Peckham	1.0	1.7	71%	1.643	(0.339 – 7.962)	0.54
Camberwell	1.0	0.8	-25%	1.041	(0.101 – 10.692)	0.97
**Control**	10.5					

No intervention was implemented in the control area and therefore a mean across all financial years is displayed
**Denotes statistical significant results at the 5% level*
- Too few applications were made to perform regression analysis

a. Licence applications received and (in other models) issued according to area, reported by outlet type, analysed using Poisson regression. These models are all adjusted for financial year, and for geographical area, including both in the model as categorical adjustment variables. The number of applications and issued licensed pre-intervention was used as the reference group (IRR = 1.00).

For peer review only

DISCUSSION

Statement of principal findings

Across the three intervention areas, absolute numbers of issued licenses for drinking establishments decreased and remained low in frequency following CIZ establishment. In Camberwell no single application for a new drinking establishment was made post intervention. Conversely, the number of new eateries increased year-on-year. The average number of issued licenses for drinking establishments, off-sales and other outlets decreased following CIZ establishment, except for off-sales in Camberwell and other outlets in Peckham. The mean number of new eateries and new takeaways increased in all areas tested. Regression analyses of outlet type and intervention showed a significant positive association for new eateries across all CIZs and for new eateries in Peckham CIZ, compared to the control area. For takeaways there was a significant increase in the number of issued licenses both across all CIZs and for Bankside CIZ, compared to the control area.

Strengths and weaknesses in relation to other studies and policy implications

The findings from this study support those from previous research that CIZs can be used as a tool to shape local alcohol environments [16], yet this is the first quantitative evidence to substantiate the theories. This is best exemplified by Peckham CIZ which was established to tackle late-night anti-social behaviour occurring due to increasing numbers of drinking establishments in the local area. Food-led establishments were exempted from the CIZ policy in Peckham. Following CIZ establishment the area has experienced a significant and substantial increase in licensed eateries.

Statements of Licensing Policy are an opportunity for LAs to set the licensing goals of urban areas *a priori*. Excluding eateries from the CIZ policy in Peckham was successful in drawing new food-led establishments to that area. Although not explicitly stated in Southwark's SOLP, Camberwell CIZ was established to tackle issues relating to street drinking. Yet the number of new off-licences in Camberwell appeared to increase following CIZ establishment and there were no new applications for drinking establishments in Camberwell post intervention. In Bankside CIZ a non-significant reduction in new Drinking Establishments was experienced in the area. Although, this is in line with the goals of the Bankside policy, a significant increase in new licensed Takeaways is unlikely to be. These findings imply that descriptive analysis of the absolute numbers of newly licensed premises in a particular geographic location is inadequate. A robust statistical test, which compares intervention areas to a control region, is required to fully appreciate the impact of CIZ policy. The results from this study can be used to inform the renewal of Southwark's SOLP and provide an

For peer review only - http://bmjopen.bmj.com/site/about/guidelines.xhtml

evidence-base from which licensing goals to diversify the night-time economy can be developed.

Strengths, weaknesses and further work

Stratifying the dataset by financial year, geographic area and outlet type rendered some strata with very few, or in some cases zero, counts. This was particularly an issue for Peckham CIZ and Camberwell CIZ, which accounted for only 7.6% and 4.4% of the data respectively. Statistical power was therefore constrained resulting in an increased risk of type two error.

Aggregation of outlet type into five discrete categories was a subjective task. Although the approach taken was as systematic as possible, and a sample of venues was sense checked against their online descriptions, there remains the possibility for interpretation. For example: an outlet which operates as a café in the daytime, and a bar at night could be categorised as either 'drinking establishment', 'eatery' or even 'other outlet'. To ensure reproducibility of the results, two researchers completed the categorisation process independently, before a final list was agreed.

Limiting outlet type to only five categories was restrictive and may have reduced precision, but necessary to provide suitable observations in each category. Categorising small drinking establishments that, for example offer food, provide seating areas for their customers and close at 23:00, as equal to a 1000-person capacity nightclub with vertical drinking until 6am is potentially problematic. Policy makers may unnecessarily restrict the former type of venue as a consequence. To better identify the types of venues that cause the most harm, further work should consider other methods of stratifying outlet type, for example by opening time and venue capacity.

A number of gaps exist in the literature preventing policy makers from making informed recommendations relating to alcohol-availability interventions. Evidence for alcohol-availability polices are from aggregate studies. Given the heterogeneous nature of the outlet types within on- and off-licence categories, it has been recommended that aggregate studies are discontinued. [9] This study provides a quantitative evaluation of CIZs within a major metropolitan area that disaggregates outlet type into five categories. This novel approach exemplifies how local policy can be used to support certain licensing goals and can facilitate the diversification of the night-time economy.

This research provides deeper insight as to the nature of alcohol exposure in different locations within an urban centre. Further work is required to understand which outlet types are associated with the greatest amount of alcohol-related harm.

For peer review only - http://bmjopen.bmj.com/site/about/guidelines.xhtml

Southwark is an inner London Borough with a thriving night-time economy. We argue these findings from this research are generalizable to similar urban centres both in the UK and internationally. The methodology employed is reproducible and scalable. Further research should attempt to replicate similar results in other LAs. In order to achieve higher statistical power, similar studies across a cluster of LA, or on a regional or national scale are recommended.

Conclusions

Cumulative Impact Zones can be used as policy levers to shape local alcohol environments to support the licensing goals of specific geographical areas and diversify the night-time economy. While we found no evidence that CIZs limit the density of different types of licensed venues, CIZs can be used as a means to encourage certain types of outlet over others and thereby change the tone of an area's night time economy. To further support the development of local licensing policies, an evidence-base for the diversification of the night-time economy is needed, and further work to understand the impact of different outlet types on alcohol-related harm outcomes is required.

For peer review only

Figure Legends

Figure 1: London Borough of Southwark's three CIZs (inset: Map of the 33 London boroughs with the London Borough of Southwark highlighted).

Figure 2: Number of issued licences per financial year in CIZ areas, by outlet type.
*Date of CIZ establishment, Peckham and Camberwell.
**Date of CIZ establishment, Bankside.

Supplementary Content

Supplementary Table 2: Area and estimated population for Southwark's three CIZs, the control region and the London Borough of Southwark

Supplementary Table 2: Number of licence applications and percentage issued in each CIZ and the control group by financial year. Data are stratified by outlet type (five categories).

Acknowledgements

The authors would like to acknowledge Southwark Council's Licensing Authority for providing access to the data for this study and for providing valuable context surrounding the local licensing policy.

Funding

This work was supported by the National Institute for Health Research (NIHR) under the Collaborations for Leadership in Applied Health Research and Care (CLAHRC) programme for North West London. The views expressed in this publication are those of the author(s) and not necessarily those of the NHS, the NIHR or the Department of Health.

Conflict of Interest

None.

Contributors

CS completed the literature review for this study, led on the data analysis and drafted the manuscript. DF provided context as to the local licensing policy. HW supported the development of the statistical model. CW helped with some of the data manipulation, created figure 1 and sourced the data for Supplementary Table 1. RP critically reviewed the manuscript and revised a second draft. HW, AP and RP inputted into the data interpretation and revised the final manuscript. All authors have approved the final manuscript.

For peer review only

For peer review only - http://bmjopen.bmj.com/site/about/guidelines.xhtml

Data Sharing Statement

No additional data are available.

For peer review only

For peer review only - http://bmjopen.bmj.com/site/about/guidelines.xhtml

Reference List

[1] Barbor T, Caetano R, Casswell S, et al, Alcohol: no ordinary commodity - research and public policy, Oxofrd Unversity Press, 2003.

[2] NHS Digital, Health Survey for England, 2016. [Available from: www.digital.nhs.uk], 2017.

[3] Burton R, Henn C, Lavoie D, O'Connor R, Perkins C, Sweeney K, Greaves F, Ferguson B, Beynon C, Belloni A, Musto V. The public health burden of alcohol and the effectiveness and cost-effectiveness of alcohol control policies: an evidence review. 2016.

[4] Campbell CA, Hahn RA, Elder R, Brewer R, Chattopadhyay S, Fielding J, Naimi TS, Toomey T, Lawrence B, Middleton JC, Task Force on Community Preventive Services. The effectiveness of limiting alcohol outlet density as a means of reducing excessive alcohol consumption and alcohol-related harms. American journal of preventive medicine. 2009 ;37(6):556-69.

[5] Jackson R, Johnson M, Campbell F, Messina J, Guillaume L, Meier PS, Goyder E, Chilcott J, Payne N. Interventions on control of alcohol price, promotion and availability for prevention of alcohol use disorders in adults and young people. London: The University of Sheffield, for NICE Centre for Public Health Excellence. 2010

[6] Stockwell T, Gruenewald PJ. Controls on the physical availability of alcohol. The essential handbook of treatment and prevention of alcohol problems. 2004 Feb 6:213-33.

[7] Institute of Alcohol Studies. Alcohol consumption factsheet 2013. [Accessed 09/08/2016]. Available from: http://www.ias.org.uk/.

[8] Richardson EA, Shortt NK, Pearce J, Mitchell R. Alcohol-related illness and deaths in Scottish neighbourhoods: is there a relationship with the number of alcohol outlets. Edinburgh: Centre for Research on Environment, Society and Health and Alcohol Focus Scotland. 2014 Oct 2.

For peer review only

[9] Gmel G, Holmes J, Studer J. Are alcohol outlet densities strongly associated with alcohol-related outcomes? A critical review of recent evidence. Drug and alcohol review. 2016 Jan;35(1):40-54.

[10] Licensing Act 2003. England and Wales. London: HMSO 2003

[11] Sharpe CA, Poots AJ, Watt H, Franklin D, Pinder RJ. Controlling alcohol availability through local policy: an observational study to evaluate Cumulative Impact Zones in a London borough. Journal of Public Health. 2017 Dec 11.

[12] Home Office. Revised Guidance issued under section 182 of the Licensing Act 2003. London: HMSO 2015

[13] Home Office. Alcohol and late night refreshment licensing England and Wales. National Statistics 2016.

[14] Humphreys DK, Smith DM. Alcohol licensing data: why is it an underused resource in public health?. Health & place. 2013 Nov 1;24:110-4.

[15] Grace D, Egan M, Lock K. Examining local processes when applying a cumulative impact policy to address harms of alcohol outlet density. Health & place. 2016 Jul 1;40:76-82.

[16] Egan M, Brennan A, Buykx P, De Vocht F, Gavens L, Grace D, Halliday E, Hickman M, Holt V, Mooney JD, Lock K. Local policies to tackle a national problem: Comparative qualitative case studies of an English local authority alcohol availability intervention. Health & place. 2016 Sep 1;41:11-8.

[17] Greater London Authority, Culture and the night-time economy. London, GLA, 2017.

[18] C. Williamson, Overview of Southwark's Population. Southwark's JSNA. Southwark Council: London. 2018.

[19] Civica. Civica Community Protection Overview 2016. [Accessed 23/07/2016]. Available from: https://www.civica.co.uk/public-protection/overview.

[20] Pitney Bowes. Data Sheet: MapInfo Location Intelligence [Available from: www.mapinfo.com Accessed 21/07/2016.

[21] Bland M. An introduction to medical statistics. Oxford University Press (UK); 2015.

For peer review only

[22] Stata Statistical Software [program]. vol. 13 version. College Station: TX:, 13 version. College Station: TX:.

For peer review only

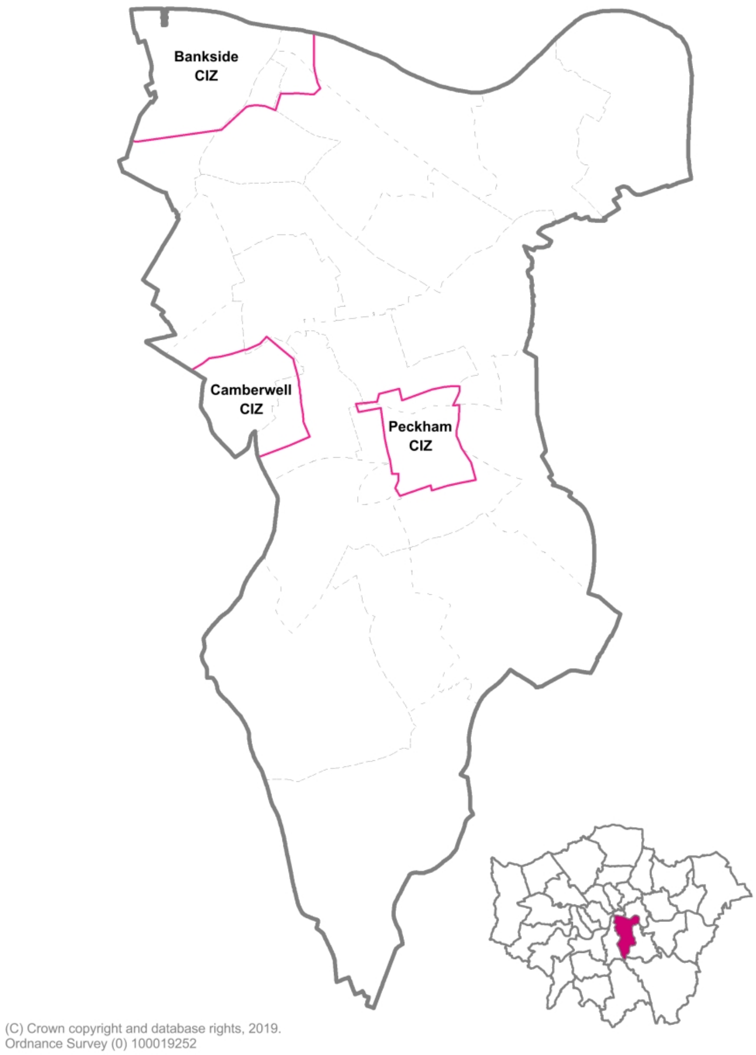

London Borough of Southwark's three CIZs (inset: Map of the 33 London boroughs with the London Borough of Southwark highlighted).

210x296mm (300 x 300 DPI)

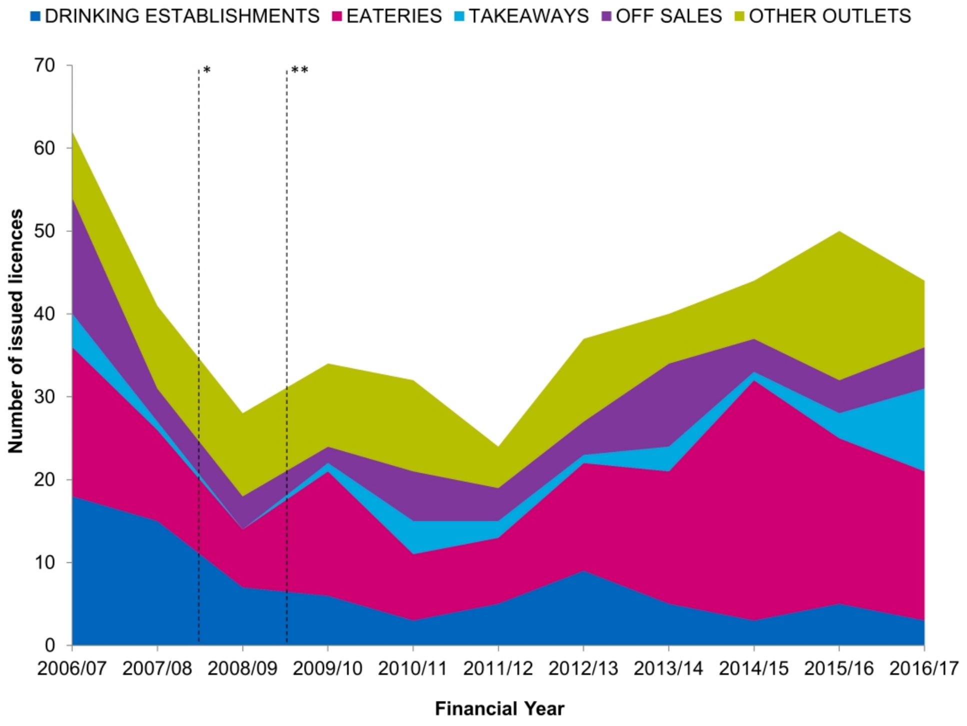

Figure 2: Number of issued licences per financial year in CIZ areas, by outlet type.
*Date of CIZ establishment, Peckham and Camberwell.
**Date of CIZ establishment, Bankside.

Supplementary Material

Supplementary Table 1: Area and estimated population for Southwark's three CIZs, the control region and the London Borough of Southwark

	AREA (km^2)	ESTIMATED TOTAL POPULATION (2016)**	
		Minimum	Maximum
**Bankside**	1.69	15,214	16,018
**Peckham**	0.91	11,226	13,820
**Camberwell**	0.74	6,518	8,474
**Control***	26.47	273,343	278,697
**Southwark**	29.81	311,655	

*The control region relates to the area of Southwark that is not a CIZ
**Southwark's CIZs are non-standard geographies: their boundaries do not align with Output Areas (OA). Output Areas are the lowest level of geography produced across all census topics. The average population in England and Wales on 27 March 2011 was 309 in OAs. Therefore, each CIZ population can only be estimated. The minimum population estimate relates the total population of all OAs that fully fit within the CIZ boundary. The maximum estimate relates the to the total population of all the OAs that fall fully within the CIZ boundary as well as those that are partially within the CIZ boundary. [1]

[1] Office for National Statistics, 2011 Census: Population and Household Estimates for Small Areas in England and Wales, March 2011, [Available from www.ons.gov.uk], 2012

For peer review only

Supplementary Table 2: Number of licence applications and percentage issued in each CIZ and the control group by financial year. Data are stratified by outlet type (five categories).

	2006/07	2007/08	2008/09	2009/10	2010/11	2011/12	2012/13	2013/14	2014/15	2015/16	2016/17	Total
**DRINKING ESTABLISHMENTS**												
**All CIZs**	**21**	**17**	**7**	**6**	**4**	**5**	**11**	**5**	**3**	**5**	**5**	**89**
% Issued	85.7	88.2	100	100	75.0	100	81.8	100	100	100	60.0	88.8
Bankside	**11**	**11**	**5**	**4**	**4**	**5**	**10**	**3**	**2**	**5**	**3**	**63**
% Issued	100	90.9	100	100	75.0	100	80.0	100	100	100	66.7	92.1
Peckham	**3**	**3**	**2**	**2**	**0**	**0**	**1**	**2**	**1**	**0**	**2**	**16**
% Issued	100	100	100	100	-	-	100	100	100	-	50.0	93.8
Camberwell	**7**	**3**	**0**	**0**	**0**	**0**	**0**	**0**	**0**	**0**	**0**	**10**
% Issued	57.1	66.7	-	-	-	-	-	-	-	-	-	60.0
**Control**	**47**	**19**	**11**	**3**	**6**	**8**	**6**	**6**	**15**	**7**	**20**	**148**
% Issued	91.5	73.7	81.8	66.7	83.3	75.0	100	83.3	80.0	85.7	85.0	84.5
**EATERIES**												
**All CIZs**	**18**	**12**	**9**	**15**	**8**	**8**	**14**	**18**	**29**	**21**	**20**	**172**
% Issued	100	91.7	77.8	100	100	100	92.9	88.9	100	95.2	90.0	94.8
Bankside	**15**	**9**	**6**	**10**	**4**	**6**	**9**	**9**	**17**	**16**	**13**	**114**
% Issued	100	88.9	83.3	100	100	100	88.9	88.9	100	93.8	84.6	93.9
Peckham	**1**	**0**	**2**	**3**	**2**	**1**	**5**	**5**	**7**	**5**	**3**	**34**
% Issued	100	0.0	50.0	100	100	100	100	80.0	100	100	100.0	94.1
Camberwell	**2**	**3**	**1**	**2**	**2**	**1**	**0**	**4**	**5**	**0**	**4**	**24**
% Issued	100	100	100	100	100	100	-	100	100	-	100	100
**Control**	**49**	**17**	**15**	**14**	**18**	**19**	**13**	**21**	**19**	**31**	**39**	**255**
% Issued	93.9	94.1	93.3	85.7	94.4	100	100	85.7	84.2	77.4	94.9	91.0
**TAKEAWAYS**												
**All CIZs**	**4**	**1**	**0**	**2**	**4**	**2**	**1**	**3**	**1**	**3**	**11**	**32**
% Issued	100	100	-	50.0	100	100	100	100	100	100	90.9	93.8
Bankside	**3**	**0**	**0**	**1**	**3**	**2**	**1**	**0**	**0**	**2**	**6**	**18**
% Issued	100	-	-	100	100	100	100	-	-	100	100	100
Peckham	**0**	**1**	**0**	**1**	**0**	**0**	**0**	**1**	**1**	**1**	**4**	**9**
% Issued	-	100	-	0	-	-	-	100	100	100	75.0	77.8
Camberwell	**1**	**0**	**0**	**0**	**1**	**0**	**0**	**2**	**0**	**0**	**1**	**5**
% Issued	100	-	-	-	100	-	-	100	-	-	100	100
**Control**	**19**	**6**	**2**	**1**	**1**	**6**	**1**	**4**	**3**	**5**	**7**	**55**
% Issued	100	83.3	100	100	100	66.7	100	100	100	100	71.4	90.9
**OFF-SALES**												
**All CIZs**	**14**	**5**	**6**	**2**	**7**	**6**	**4**	**11**	**4**	**4**	**5**	**68**
% Issued	100	80.0	66.7	100	85.7	66.7	100	90.9	100	100	100	89.7
Bankside	**7**	**3**	**5**	**1**	**5**	**1**	**1**	**5**	**4**	**3**	**4**	**39**
% Issued	100	100	60.0	100	100	100	100	100	100	100	100	94.9
Peckham	**5**	**1**	**0**	**1**	**2**	**2**	**3**	**2**	**0**	**1**	**1**	**18**
% Issued	100.0	-	-	100	50.0	-	100	50.0	-	100	100	72.2
Camberwell	**2**	**1**	**1**	**0**	**0**	**3**	**0**	**4**	**0**	**0**	**0**	**11**
% Issued	100	100	100	-	-	100	-	100	-	-	-	100

**Control**	**40**	**18**	**15**	**11**	**9**	**17**	**10**	**18**	**16**	**16**	**10**	**180**
% Issued	92.5	94.4	86.7	81.8	100	100	100	100	81.3	68.8	70.0	89.4
						**OTHER OUTLETS**						
**All CIZs**	**9**	**10**	**12**	**12**	**12**	**6**	**10**	**9**	**8**	**21**	**11**	**120**
% Issued	88.9	100	83.3	83.3	91.7	83.3	100	66.7	87.5	85.7	72.7	85.8
Bankside	**7**	**10**	**10**	**12**	**9**	**5**	**7**	**6**	**7**	**15**	**9**	**97**
% Issued	85.7	100	90.0	83.3	100	80.0	100	66.7	100	80.0	77.8	87.6
Peckham	**1**	**0**	**2**	**0**	**2**	**1**	**3**	**3**	**0**	**5**	**1**	**18**
% Issued	100	-	50.0	0.0	50.0	100	100	66.7	-	100	0.0	77.8
Camberwell	**1**	**0**	**0**	**0**	**1**	**0**	**0**	**0**	**1**	**1**	**1**	**5**
% Issued	100	-	-	-	100	-	-	-	0.0	100	100	80.0
**Control**	**18**	**6**	**5**	**12**	**8**	**10**	**10**	**11**	**19**	**20**	**16**	**135**
% Issued	94.4	100	60.0	83.3	100	100	100	100	78.9	80.0	62.5	85.9

For peer review only

STROBE 2007 (v4) checklist of items to be included in reports of observational studies in epidemiology*

Checklist for cohort, case-control, and cross-sectional studies (combined)

Section/Topic	Item #	Recommendation	Reported on page
**Title and abstract**	1	(*a*) Indicate the study's design with a commonly used term in the title or the abstract	1
		(*b*) Provide in the abstract an informative and balanced summary of what was done and what was found	2
**Introduction**			
Background/rationale	2	Explain the scientific background and rationale for the investigation being reported	4-6
Objectives	3	State specific objectives, including any pre-specified hypotheses	5
**Methods**			
Study design	4	Present key elements of study design early in the paper	2
Setting	5	Describe the setting, locations, and relevant dates, including periods of recruitment, exposure, follow-up, and data collection	2 and 7
Participants	6	(*a*) *Cohort study*—Give the eligibility criteria, and the sources and methods of selection of participants. Describe methods of follow-up<br>*Case-control study*—Give the eligibility criteria, and the sources and methods of case ascertainment and control selection. Give the rationale for the choice of cases and controls<br>*Cross-sectional study*—Give the eligibility criteria, and the sources and methods of selection of participants	Data source is explained on page (this is data that wa readily available)
		(*b*) *Cohort study*—For matched studies, give matching criteria and number of exposed and unexposed<br>*Case-control study*—For matched studies, give matching criteria and the number of controls per case	N/A
Variables	7	Clearly define all outcomes, exposures, predictors, potential confounders, and effect modifiers. Give diagnostic criteria, if applicable	7
Data sources/ measurement	8*	For each variable of interest, give sources of data and details of methods of assessment (measurement). Describe comparability of assessment methods if there is more than one group	7
Bias	9	Describe any efforts to address potential sources of bias	7 (two adjustmen variables were included in the statistical model)
Study size	10	Explain how the study size was arrived at	7 (all licence applications within defined time perio were included, a study sample was therefore not

For peer review only

			necessary)
Quantitative variables	11	Explain how quantitative variables were handled in the analyses. If applicable, describe which groupings were chosen and why	7
Statistical methods	12	(*a*) Describe all statistical methods, including those used to control for confounding	7
		(*b*) Describe any methods used to examine subgroups and interactions	7
		(*c*) Explain how missing data were addressed	N/A (a comprehensive dat set was sourced)
		(*d*) *Cohort study*—If applicable, explain how loss to follow-up was addressed<br>*Case-control study*—If applicable, explain how matching of cases and controls was addressed<br>*Cross-sectional study*—If applicable, describe analytical methods taking account of sampling strategy	N/A
		(*e*) Describe any sensitivity analyses	N/A
**Results**			
Participants	13*	(a) Report numbers of individuals at each stage of study—eg numbers potentially eligible, examined for eligibility, confirmed eligible, included in the study, completing follow-up, and analysed	N/A
		(b) Give reasons for non-participation at each stage	N/A
		(c) Consider use of a flow diagram	N/A
Descriptive data	14*	(a) Give characteristics of study participants (eg demographic, clinical, social) and information on exposures and potential confounders	N/A
		(b) Indicate number of participants with missing data for each variable of interest	7
		(c) *Cohort study*—Summarise follow-up time (eg, average and total amount)	N/A
Outcome data	15*	*Cohort study*—Report numbers of outcome events or summary measures over time	10, 11-12 (see also supplementary material)
		Case-control study—Report numbers in each exposure category, or summary measures of exposure	10, 11-12 (see also supplementary material)
		Cross-sectional study—Report numbers of outcome events or summary measures	10, 11-12 (see also supplementary material)
Main results	16	(*a*) Give unadjusted estimates and, if applicable, confounder-adjusted estimates and their precision (eg, 95% confidence interval). Make clear which confounders were adjusted for and why they were included	11-12
		(*b*) Report category boundaries when continuous variables were categorized	N/A
		(*c*) If relevant, consider translating estimates of relative risk into absolute risk for a meaningful time period	N/A

For peer review only

Other analyses	17	Report other analyses done—eg analyses of subgroups and interactions, and sensitivity analyses	10, 11-12
**Discussion**			
Key results	18	Summarise key results with reference to study objectives	13
Limitations	19	Discuss limitations of the study, taking into account sources of potential bias or imprecision. Discuss both direction and magnitude of any potential bias	13-14
Interpretation	20	Give a cautious overall interpretation of results considering objectives, limitations, multiplicity of analyses, results from similar studies, and other relevant evidence	13-15
Generalisability	21	Discuss the generalisability (external validity) of the study results	15
**Other information**			
Funding	22	Give the source of funding and the role of the funders for the present study and, if applicable, for the original study on which the present article is based	16

*Give information separately for cases and controls in case-control studies and, if applicable, for exposed and unexposed groups in cohort and cross-sectional studies.
Note: An Explanation and Elaboration article discusses each checklist item and gives methodological background and published examples of transparent reporting. The STROBE checklist is best used in conjunction with this article (freely available on the Web sites of PLoS Medicine at http://www.plosmedicine.org/, Annals of Internal Medicine at http://www.annals.org/, and Epidemiology at http://www.epidem.com/). Information on the STROBE Initiative is available at www.strobe-statement.org.

For peer review only

BMJ Open

An observational study to examine how Cumulative Impact Zones influence alcohol availability from different types of licensed outlets in an inner London Borough

Journal:	*BMJ Open*
Manuscript ID	bmjopen-2018-027320.R2
Article Type:	Original research
Date Submitted by the Author:	19-Jul-2019
Complete List of Authors:	Sharpe, Carolyn; Imperial College London School of Public Health, Department of Primary Care and Public Health; Southwark Council, Public Health Directorate<br>Poots, Alan; Imperial College London, NIHR CLARHC Northwest London; Independent Researcher<br>Watt, Hilary; Imperial College London, Primary Care and Public Health<br>Williamson, Chris; Southwark Council, Public Health Division, Place & Wellbeing Department<br>Franklin, David; Southwark Council, Licensing<br>Pinder, Richard; Imperial College London School of Public Health, Department of Primary Care and Public Health; Southwark Council, Public Health Directorate
**Primary Subject Heading**:	Public health
Secondary Subject Heading:	Health policy, Epidemiology
Keywords:	PUBLIC HEALTH, Cumulative Impact Zone, Alcohol Licensing, Alcohol Availability

SCHOLARONE™ Manuscripts

An observational study to examine how Cumulative Impact Zones influence alcohol availability from different types of licensed outlets in an inner London Borough

Authors:

C A Sharpe, Specialty Registrar, Imperial College London & Southwark Council[1,2]
carolyn.sharpe1@nhs.net – *corresponding & first author*

Dr A J Poots, Principal Information Analyst, formerly Imperial College London[4,a]

H Watt, Consultant Statistician & Teaching Fellow, Imperial College London[1]

Chris Williamson, Head of Public Health Intelligence, Southwark Council[2]

D Franklin, Head of Licensing, Southwark Council[3]

Dr R J Pinder, Consultant in Public Health, Imperial College London & Southwark Council[1,2]

Affiliations at the time of study:

1. Department of Primary Care and Public Health, School of Public Health, Imperial College London, Reynolds Building, St Dunstans Road, London, W6 8RP, United Kingdom

2. Public Health Directorate, Southwark Council, 1st Floor, Hub 1, PO Box 64529, London, SE1P 5LX, United Kingdom

3. Licensing Team, Southwark Council, 3rd Floor, Hub 1, PO Box 64529, London, SE1P 5LX, United Kingdom

4. NIHR CLARHC Northwest London, Imperial College London, Floor 4, Lift Bank D, Chelsea and Westminster Hospital, 369 Fulham Road, London, SW10 9NH, United Kingdom

Current affiliations:

a) Independent researcher

Word Count: 3232 words
(4000 word limit)

For peer review only - http://bmjopen.bmj.com/site/about/guidelines.xhtml

Abstract (290 / 300 words)

OBJECTIVES: Cumulative Impact Zones (CIZs) are a widely implemented local policy intended to restrict alcohol availability in areas proliferated with licensed outlets. Limited previous research has questioned their effectiveness and suggested they may play a more nuanced role in shaping local alcohol environments. This study evaluates the association between CIZ implementation and the number of licence applications made and the number issued, relative to a control region.

DESIGN: A quantitative observational study.

SETTING: The inner London Borough of Southwark, which currently enforces three CIZs.

POPULATION: Licence applications received by Southwark Council's Licensing Authority between 1 April 2006 and 31 March 2017 (N = 1254).

INTERVENTIONS: CIZ implementation.

PRIMARY OUTCOME MEASURES: Five outlet types were categorised and evaluated: Drinking Establishments, Eateries, Takeaways, Off Sales, and Other Outlets. Primary outcome measures were the number of applications received and the number of licences issued. These were analysed using Poisson regression of counts over time.

RESULTS: Across all CIZs, implementation was associated with greater increases in the number of eateries in CIZ regions (IRR = 1.58, 95% CI: 1.02 – 2.52, P = 0.04) and number of takeaway venues (IRR = 3.89, 95% CI: 1.32 – 11.49, P = 0.01), relative to the control area. No discernible association was found for the remaining outlet types. Disaggregating by area indicated a 10-fold relative increase in the number of new eateries in Peckham CIZ (IRR = 10.38, 95% CI: 1.39 – 77.66, P = 0.02) and a four-fold relative increase in the number of newly licensed takeaways in Bankside CIZ (IRR = 4.38, 95% CI: 1.20 – 15.91, P = 0.03).

CONCLUSIONS: Cumulative Impact Zones may be useful as policy levers to shape local alcohol environments to support the licensing goals of specific geographical areas and diversify the night-time economy.

For peer review only - http://bmjopen.bmj.com/site/about/guidelines.xhtml

Article Summary

Strengths and limitations of this study

- This study provides the first quantitative evaluation of CIZs that disaggregates outlet type into five discrete categories.
- This research provides deeper insight in to the nature of alcohol exposure in different locations within an urban centre and therefore provides an evidence base from which local policy can be developed to support local licensing goals which may include diversification of the night-time economy.
- This study shows that descriptive analyses of alcohol control policies (using absolute numbers) are insufficient, and provides a robust statistical approach that can be employed in other urban settings.
- Although substantial steps were taken to assure the reproducibility of the results, the categorisation of outlet type into five discrete categories involved qualitative assessment and there remains a risk of subjectivity.
- Necessary stratification of the dataset rendered some strata with very few (or in some cases zero) counts, thereby constraining statistical power.

Key words

Public Health, Cumulative Impact Zone, Alcohol Licensing, Alcohol Availability

For peer review only

Main Text

INTRODUCTION

Alcohol harm and opportunities to control availability

Alcohol occupies a prominent role in modern society. It is a commodity used by many, across social strata, as a relaxant and a means of enjoyment [1]. The misuse of alcohol leads to increased morbidity and mortality. In England in 2016, it was estimated that almost 13 million adults drank at levels that increased their risk of harm. [2] Among those aged 15 to 49, alcohol has become the leading risk factor for ill-health, early mortality, and disability. [3]

Outlet spatial density is positively associated with consumption. [4] [5] Policies to restrict the physical availability of alcohol are well supported in the literature. [6] However, such evidence is based on aggregate studies that consider all alcohol-outlet types as equal. At best, outlets have been categorised as either on or off-sales. On-sales of alcohol refer to venues such as pubs, nightclubs and restaurants where alcohol is purchased to be consumed on the premises. Off-sales of alcohol refer to premises such as off-licenses and supermarkets from which alcohol is purchased to be consumed away from the retailer's premises. [7] Although greater association has been found between off-sales and alcohol-related hospitalisations and deaths than with on-sales, [8] both on- and off-licences cover a broad range of establishments. Such aggregate studies remain a crude way to evaluate alcohol availability [9] and debate remains around when to use aggregated and disaggregated analyses. [10]

A full description of the licensing process in England and Wales, as defined by the Licensing Act 2003, [11] is outlined in our previous paper. [12] In summary, Revised Guidance issued under section 182 of the Licensing Act 2003 enables local licensing authorities (LAs) to address the cumulative impact of a concentration of licensed premises. [13] Cumulative Impact is defined as "the potential impact on the promotion of the licensing objectives of a significant number of licensed premises concentrated in one area". [13] The licensing objectives, in England and Wales, as defined by the Licensing Act 2003 are: the prevention of crime and disorder; public safety; the prevention of public nuisance; and the protection of children from harm [11].

A CIZ may therefore be established, if a LA can evidentially justify that the licensing objectives are not being upheld in a particular location. This evidence must be published in a LA's Statement of Licensing Policy (SOLP). The Licensing Act 2003 requires all LAs to develop and publish a SOLP at least every five years. [11] Evidence to establish a CIZ relates to the licensing objectives. Local crime, disorder and anti-social behaviour statistics

For peer review only - http://bmjopen.bmj.com/site/about/guidelines.xhtml

For peer review only

can be evaluated alongside acute health data, such as alcohol-related ambulance attendances and hospital admissions. Environmental health complaints, particularly in relation to litter and noise, can also be included. [13]

Current evidence relating to CIZs

In 2016 there were 215 CIZs established across 106 LAs [14]. Despite their widespread establishment, there is a scarcity of literature evaluating their impact. [15] Revised Guidance issued under section 182 of the Licensing Act 2003 states that an application within a CIZ would normally be refused, or subject to limitations, unless the applicant can demonstrate there will be no cumulative impact on the licensing objectives. [13] A common perception therefore follows that CIZs limit alcohol availability in areas already saturated. Previous work undertaken in the London Borough of Southwark found no evidence that CIZ establishment reduced the number of licence applications or issued licenses across its three policy areas combined, when compared to a control region. On analysing one CIZ area in isolation, establishment was associated with a statistically significant 133% increase in successful applications. [12]

Qualitative research, conducted in 2016, investigating the drivers behind CIZ implementation has suggested them to be more nuanced than simply restricting outlet density. [16] Participants described how CIZs have evolved to encourage local regeneration goals. CIZs tended not to be established to cap the number of licenced outlets, rather they are viewed as a tool to control the temporal availability of alcohol and even to encourage a certain type of application e.g. from arts, food and coffee led establishments as opposed to 'vertical drinking' bars.

Plans to diversify night-time economies and offer alternatives to alcohol-led activities are emerging. In April 2017, the Mayor of London published guidance for culture and the night-time economy in the capital and identified diversification and inclusiveness as key areas of focus. Through encouraging leisure and cultural venues e.g.: late night markets, museums, shops, cafes, theatres, and fairs, urban centres hope to a wider clientele. Examples of which include non-drinkers, older people, families and those with a disability. [17] Despite these plans, an evidence base in support of diversifying the night-time economy is lacking.

Gaps in the literature

A key research gap exists around the assumption that all on- and off-licensed outlets are treated as equal. [9] Although aggregate studies may show an association between outlet density and harm, they are of limited use to policy makers. Interventions need to be targeted towards outlets that cause the most harm. Therefore, through stratifying by outlet type, we

For peer review only - http://bmjopen.bmj.com/site/about/guidelines.xhtml

aim to understand how CIZ establishment is associated with the number of licence applications and issued licences for different outlet types. Qualitative evidence indicates that CIZs can facilitate the proliferation of certain types of premises, thereby diversifying local night-time economies. [16] We therefore propose a quantitative methodology to test for this.

Southwark's three cumulative impact zones

Southwark is an inner London borough with an estimated population of 311,655. [18] Southwark is home to approximately 1,400 licensed premises which make a significant contribution to its culture and economy. However, some locations within the borough have reached saturation point. Within these areas of saturation, Southwark enforces three CIZs (Figure 1); Camberwell CIZ and Peckham CIZ were both established 05 November 2008. Bankside CIZ was established a year later on 05 November 2009. The implementation of each CIZ was to achieve a unique objective. (Franklin D. Head of Licensing, Southwark Council. Personal communication. 17 July 2018) With almost 70% of all CIZ licence applications falling within Bankside alone, the intervention aimed to tackle binge drinking-related issues. The Bankside CIZ policy applies to all venue types except for theatres and cinemas. Peckham and Camberwell CIZs were established to tackle late-night anti-social behaviour and street drinking respectively. Notably, food-led establishments, such as restaurants and cafes, are exempt from both CIZ policies. The size and estimated population of Southwark's CIZ areas is detailed in the supplementary information (Supplementary Table 1).

For peer review only

For peer review only - http://bmjopen.bmj.com/site/about/guidelines.xhtml

METHODS

Data collection and collation

A comprehensive alcohol licensing data set of all applications made within Southwark between 1 April 2006 and 31 March 2017 was obtained from Authority Public Protection (APP); a database used by the council to manage licence applications. [19] Licensing data prior to 2006 could not be analysed by year of application and year of issue, as in 2005 Southwark transitioned their licensing data to APP from a previous data base. Geographical information software (GIS) [20] was used to assign each outlet application an easting and northing. These coordinates were then used to identify applications within each CIZ region and the control group. The issue date was used as a proxy that applications were successful. All applications without an issue date were categorised as having been rejected or withdrawn. An outlet type field was obtained from APP containing 100 different entries for outlet type. This field was aggregated into five mutually-exclusive categories;

- Bars, public-houses, hotel bars and nightclubs were grouped and are collectively referred to as **drinking establishments**
- Restaurants and cafes were aggregated together and are collectively referred to as **eateries**
- Licensed takeaway food restaurants were kept separate and are referred to as **takeaways**
- Off-licences, grocery stores and supermarkets are grouped into one **off-sales** category
- Those alcohol-outlets that do not fit into any of the above categories are collectively referred to as **other outlets** (such as florists, boats and schools).

To ensure reproducibility of the results, two researchers completed the categorisation process independently, before a final list was agreed.

Licence applications were grouped by financial year (April to the following March) starting from 2006/07. An indicator variable for the intervention was fitted, to indicate which time periods in which regions had a CIZ in operation. The three CIZs were established on 5 November in each of the respective years. We therefore grouped applications in 365-day periods relative to the date of CIZ establishment.

Statistical analyses

Poisson regression [21] was employed to model the association between CIZ establishment (indicator variable) and firstly the number of applications and secondly the number of licenses issued. The assumptions of the Poisson model were checked, and the data were consistent with equidispersion (the mean and variance being equal). We adjusted for

For peer review only - http://bmjopen.bmj.com/site/about/guidelines.xhtml

financial yearly trend, across all regions tested, and for geographical area, including both in the model as categorical adjustment variables; financial yearly trend did not fit well enough to a linear model for that to be a viable alternative. Therefore, the resulting incidence rate ratios (IRRs) for CIZs represent ratios of changes on introduction of a CIZ into an area, relative to the changes that occur with time in all regions (in the absence of any change in CIZ status and including in control regions). An adjustment was made to accommodate for the CIZs being established mid-financial year. Statistical analyses were executed using STATA 13.0. [22] IRRs were calculated along with 95% confidence intervals (CIs) and p-values

Patient and Public Involvement

There was no patient or public involvement in this study.

For peer review only

For peer review only - http://bmjopen.bmj.com/site/about/guidelines.xhtml

RESULTS

Descriptive findings

A full breakdown of the number of licence applications, and percentage issued, in each CIZ and the control group by financial year is provided in the supplementary material (Supplementary Table 2).

From 1 April 2006 to 31 March 2017, 1254 license applications were received by Southwark Council (Table 1). The most common application type was for eateries, with 427 (34.1%) applications made over the 10 years. The number of applications for drinking establishments, off-sales and other outlets were similar; 237 (18.9%), 248 (19.8%) and 255 (20.3%) respectively. During this time period, the council received only 87 (6.9%) applications for takeaways.

Across the period and across types of venue, 38.4% of applications were made within CIZ areas. “Other outlets” (venue type) had the highest proportion of applications within CIZs (47.1%), of which over 80% were made within Bankside CIZ alone. “Off-sales” venue type had the lowest proportion of applications within CIZ areas with 72.6% relating to the control region. Over a quarter (26.4%) of all new applications were made in Bankside. Peckham and Camberwell consistently received fewer applications than Bankside across the period, and for all outlet categories.

Between 2009/10 and 2011/12, following CIZ establishment, the number of issued licenses for drinking establishments, eateries and other outlets decreases (Figure 2). Numbers for off-sales and takeaways remained relatively stable. Although the number of new drinking establishments remained low, there was a general trend upwards in the number of new eateries and “other outlets” between 2011/12 and 2016/17.

Following CIZ implementation, the mean number of applications and issued licenses for drinking establishments decreased across the three intervention areas (Table 2). The reductions were not uniform and ranged from a 36% reduction in Peckham to a 100% reduction in Camberwell. Applications and issued licenses for eateries and takeaways increased across the intervention regions. Notably, Peckham experienced a seven-fold increase in the mean number of newly issued licenses for eateries and Camberwell experienced a 50% increase. For off-sales, there was a decrease in the number of new licenses issued in both Bankside and Peckham, with Camberwell experiencing a 163% increase. Other outlets increased by 71% in Peckham, with the other two intervention areas seeing a decrease in both the mean number of applications and issued licenses.

For peer review only - http://bmjopen.bmj.com/site/about/guidelines.xhtml

Regression analysis

Multivariate regression analysis indicated that there was no discernible association between CIZ establishment (adjusted for overall time trends across all regions) and the number of applications and issued licenses for drinking establishments across all CIZs and for each CIZ region individually (Table 2). For eateries, there is some evidence that CIZ implementation was associated with a 58% increase in the number of licenses issued (IRR = 1.58, 95% CI: 1.02 – 2.52, P = 0.04). This association is driven by a 937% increase in the number of new eateries in Peckham (IRR = 10.38, 95% CI: 1.39 – 77.66, P = 0.02). There was some evidence for an increase in the number of applications and issued licenses for takeaways across the three intervention regions, adjusted for overall time trends across all regions (IRR = 3.57, 95% CI: 1.23 – 10.24, P = 0.02) and (IRR = 3.89, 95% CI: 1.32 – 11.49, P = 0.01) respectively. On disaggregating the areas, there was only appreciable evidence for the number of newly issued takeaways in Bankside (IRR = 4.38, 95% CI: 1.20 – 15.91, P = 0.03). For off-sales and other outlets, there was little evidence for associations between CIZ establishment and the number of applications and issued licenses across all CIZs. Camberwell CIZ experienced a substantial relative increase in the number of off-sale applications (IRR = 3.65, 95% CI: 1.00 – 13.30, P = 0.05), but with only weak evidence for this association. Bankside CIZ shows some evidence for a decrease in the number of applications for other outlets (IRR = 0.56, 95% CI: 0.33 – 0.95), P = 0.03); however other regions reported relative decreases, albeit with no ability to rule out random chance as an explanation.

.

For peer review only

For peer review only - http://bmjopen.bmj.com/site/about/guidelines.xhtml

Table 1: Number of licence applications made within each CIZ and the control group, by outlet type with percentages indicating, for each licence type, the proportion in the CIZ areas or the control area, 2006/07 – 2015/16.

	Drinking Establishments		Eateries		Takeaways		Off-sales		Other Outlets		TOTAL	
	n	%	n	%	n	%	n	%	n	%	n	%
**All CIZs**	**89**	**37.6**	**172**	**40.3**	**32**	**36.8**	**68**	**27.4**	**120**	**47.1**	**481**	**38.4**
Bankside	*63*		*114*		*18*		*39*		*97*		***331***	
Peckham	*16*		*34*		*9*		*18*		*18*		***95***	
Camberwell	*10*		*24*		*5*		*11*		*5*		***55***	
**Control**	**148**	**62.4**	**255**	**59.7**	**55**	**63.2**	**180**	**72.6**	**135**	**52.9**	**773**	**61.6**
**TOTAL [%]**	**237**	**[18.9]**	**427**	**[34.1]**	**87**	**[6.9]**	**248**	**[19.8]**	**255**	**[20.3]**	**1254**	**[100]**

For peer review only

For peer review only - http://bmjopen.bmj.com/site/about/guidelines.xhtml

Table 2: Before and after analysis of Southwark's three CIZs by outlet type, 2006/07 – 2015/16. IRR refers to incidence rate ratios in CIZ region(s) relative to changes over time, allowing for region.

LICENSE APPLICATIONS AND ISSUED LICENSES BY VENUE TYPE						
				Adjusted model [a]		
	**Pre-CIZ** Mean per financial year	**Post-CIZ** Mean per financial year	**Change**	**IRR**	**(95%CI)**	**p-value**
DRINKING ESTABLISHMENTS						
**Number of applications**						
**All CIZs**	11.5	4.7	-59%	1.433	(0.837 – 2.455)	0.19
Bankside	7.3	4.3	-41%	1.340	(0.745 – 2.411)	0.33
Peckham	2.3	1.5	-36%	1.704	(0.601 – 4.834)	0.32
Camberwell	5.0	0	-100%	-	-	-
**Control**	13.5					
**Number of issued licences**						
**All CIZs**	10.3	4.3	-58%	1.344	(0.763 – 2.368)	0.31
Bankside	7.0	3.8	-46%	1.253	(0.675 – 2.328)	0.47
Peckham	2.3	1.3	-43%	1.556	(0.530 – 4.569)	0.42
Camberwell	3.0	0	-100%	-	-	-
**Control**	8.1					
EATERIES						
**Number of applications**						
**All CIZs**	10.8	12.6	17%	1.484	(0.972 – 2.266)	0.07
Bankside	8.8	9.9	13%	1.223	(0.767 – 1.951)	0.40
Peckham	1.0	3.6	256%	5.083	(1.185 – 21.803)	0.03
Camberwell	2.0	3.0	50%	1.869	(0.712 – 4.905)	0.20
**Control**	23.2					
**Number of issued licences**						
**All CIZs**	10.0	12.1	21%	1.582	(1.020 – 2.452)	0.04
Bankside	8.3	9.3	12%	1.260	(0.777 – 2.041)	0.35
Peckham	0.5	3.4	589%	10.377	(1.386 – 77.664)	0.02
Camberwell	2.0	3.0	50%	1.916	(0.727 – 5.043)	0.18
**Control**	21.1					
TAKEAWAYS						
**Number of applications**						
**All CIZs**	2.0	2.1	7%	3.571	(1.225 – 10.242)	0.02
Bankside	2.0	2.8	40%	3.500	(0.981 – 12.492)	0.05
Peckham	1.0	1.6	60%	2.400	(0.248 – 23.236)	0.45
Camberwell	1.0	1.3	33%	6.330	(0.660 – 63.639)	0.12
**Control**	5.0					
**Number of issued licences**						
**All CIZs**	2.0	2.0	0%	3.894	(1.320 – 11.492)	0.01

For peer review only - http://bmjopen.bmj.com/site/about/guidelines.xhtml

Bankside	2.0	2.8	40%	4.375	(1.203 – 15.911)	0.03
Peckham	1.0	1.2	20%	1.667	(0.161 – 17.257)	0.67
Camberwell	1.0	1.3	33%	7.600	(0.746 – 77.431)	0.09
**Control**	4.5					
OFF-SALES						
**Number of applications**						
**All CIZs**	8.3	5.4	-36%	1.393	(0.773 – 2.510)	0.27
Bankside	5.0	3.0	-40%	1.092	(0.536 – 2.221)	0.81
Peckham	3.0	1.7	-43%	1.275	(0.453 – 3.584)	0.64
Camberwell	1.3	3.5	163%	3.650	(1.002 – 13.298)	0.05
**Control**	16.4					
**Number of issued licences**						
**All CIZs**	7.3	4.9	-34%	1.535	(0.824 – 2.861)	0.18
Bankside	4.3	3.0	-31%	1.316	(0.625 – 2.769)	0.47
Peckham	2.5	1.1	-54%	1.067	(0.331 – 3.434)	0.91
Camberwell	1.3	3.5	163%	3.350	(0.918 – 12.223)	0.07
**Control**	14.6					
OTHER OUTLETS						
**Number of applications**						
**All CIZs**	10.0	6.9	-31%	0.623	(0.371 – 1.044)	0.07
Bankside	9.3	7.5	-19%	0.556	(0.325 – 0.951)	0.03
Peckham	1.0	2.3	129%	2.171	(0.466 – 10.121)	0.32
Camberwell	1.0	1.0	0%	1.143	(0.120 – 10.876)	0.91
**Control**	12.3					
**Number of issued licences**						
**All CIZs**	9.0	5.9	-35%	0.601	(0.346 – 1.047)	0.07
Bankside	8.3	6.5	-21%	0.571	(0.321 – 1.013)	0.55
Peckham	1.0	1.7	71%	1.643	(0.339 – 7.962)	0.54
Camberwell	1.0	0.8	-25%	1.041	(0.101 – 10.692)	0.97
**Control**	10.5					

No intervention was implemented in the control area and therefore a mean across all financial years is displayed
- = Too few applications were made to perform regression analysis

a. Licence applications received and (in other models) issued according to area, reported by outlet type, analysed using Poisson regression. These models are all adjusted for financial year, and for geographical area, including both in the model as categorical adjustment variables. The number of applications and issued licensed pre-intervention was used as the reference group (IRR = 1.00).

For peer review only

DISCUSSION

Statement of principal findings

Across the three intervention areas, absolute numbers of issued licenses for drinking establishments decreased and remained low in frequency following CIZ establishment. In Camberwell no single application for a new drinking establishment was made post intervention. Conversely, the number of new eateries increased year-on-year. The average number of issued licenses for drinking establishments, off-sales and other outlets decreased following CIZ establishment, except for off-sales in Camberwell and other outlets in Peckham. The mean number of new eateries and new takeaways increased in all areas tested. Regression analyses of outlet type and intervention gave some evidence for a positive association for new eateries across all CIZs and for new eateries in Peckham CIZ, compared to the control area. For takeaways there was some evidence for an increase in the number of issued licenses both across all CIZs and for Bankside CIZ, compared to the control area.

Strengths and weaknesses in relation to other studies and policy implications

The findings from this study support those from previous research that CIZs can be used as a tool to shape local alcohol environments [16], yet this is the first quantitative evidence to substantiate the theories. This is best exemplified by Peckham CIZ which was established to tackle late-night anti-social behaviour occurring due to increasing numbers of drinking establishments in the local area. Food-led establishments were exempted from the CIZ policy in Peckham. We found some evidence the area experienced a substantial increase in licensed eateries following CIZ establishment.

Statements of Licensing Policy are an opportunity for LAs to set the licensing goals of urban areas *a priori*. Excluding eateries from the CIZ policy in Peckham was successful in drawing new food-led establishments to that area. Although not explicitly stated in Southwark's SOLP, Camberwell CIZ was established to tackle issues relating to street drinking. Yet the number of new off-licences in Camberwell appeared to increase following CIZ establishment and there were no new applications for drinking establishments in Camberwell post intervention. In Bankside CIZ a reduction in new Drinking Establishments was experienced. Although, this is in line with the goals of the Bankside policy, an increase in new licensed Takeaways is unlikely to be. These findings imply that descriptive analysis of the absolute numbers of newly licensed premises in a particular geographic location is inadequate. A robust statistical test, which compares intervention areas to a control region, is required to fully appreciate the impact of CIZ policy. The results from this study can be used to inform

For peer review only - http://bmjopen.bmj.com/site/about/guidelines.xhtml

the renewal of Southwark's SOLP and provide an evidence-base from which licensing goals to diversify the night-time economy can be developed.

Strengths, weaknesses and further work

Stratifying the dataset by financial year, geographic area and outlet type rendered some strata with very few, or in some cases zero, counts. This was particularly an issue for Peckham CIZ and Camberwell CIZ, which accounted for only 7.6% and 4.4% of the data respectively. Statistical power was therefore constrained resulting in an increased risk of type two error.

Aggregation of outlet type into five discrete categories was a subjective task. Although the approach taken was as systematic as possible, and a sample of venues was sense checked against their online descriptions, there remains the possibility for interpretation. For example: an outlet which operates as a café in the daytime, and a bar at night could be categorised as either 'drinking establishment', 'eatery' or even 'other outlet'. To ensure reproducibility of the results, two researchers completed the categorisation process independently, before a final list was agreed.

Limiting outlet type to only five categories was restrictive and may have reduced precision, but necessary to provide suitable observations in each category. Categorising small drinking establishments that, for example offer food, provide seating areas for their customers and close at 23:00, as equal to a 1000-person capacity nightclub with vertical drinking until 6am is potentially problematic. Policy makers may unnecessarily restrict the former type of venue as a consequence. To better identify the types of venues that cause the most harm, further work should consider other methods of stratifying outlet type, for example by opening time and venue capacity.

A number of gaps exist in the literature preventing policy makers from making informed recommendations relating to alcohol-availability interventions. Evidence for alcohol-availability polices are from aggregate studies. Given the heterogeneous nature of the outlet types within on- and off-licence categories, studies providing a disaggregated analysis are needed. This study provides a quantitative evaluation of CIZs within a major metropolitan area that disaggregates outlet type into five categories. This novel approach exemplifies how local policy can be used to support certain licensing goals and can facilitate the diversification of the night-time economy.

This research provides deeper insight as to the nature of alcohol exposure in different locations within an urban centre. Further work is required to understand which outlet types are associated with the greatest amount of alcohol-related harm.

For peer review only - http://bmjopen.bmj.com/site/about/guidelines.xhtml

Southwark is an inner London Borough with a thriving night-time economy. We argue these findings from this research are generalizable to similar urban centres both in the UK and internationally. The methodology employed is reproducible and scalable. Further research should attempt to replicate similar results in other LAs. In order to achieve higher statistical power, similar studies across a cluster of LA, or on a regional or national scale are recommended.

Conclusions

Cumulative Impact Zones can be used as policy levers to shape local alcohol environments to support the licensing goals of specific geographical areas and diversify the night-time economy. While we found no evidence that CIZs limit the density of different types of licensed venues, CIZs may be used as a means to encourage certain types of outlet over others and thereby change the tone of an area's night time economy. To further support the development of local licensing policies, an evidence-base for the diversification of the night-time economy is needed, and further work to understand the impact of different outlet types on alcohol-related harm outcomes is required.

For peer review only

Figure Legends

Figure 1: London Borough of Southwark's three CIZs (inset: Map of the 33 London boroughs with the London Borough of Southwark highlighted).

Figure 2: Number of issued licences per financial year in CIZ areas, by outlet type.
*Date of CIZ establishment, Peckham and Camberwell.
**Date of CIZ establishment, Bankside.

Supplementary Content

Supplementary Table 1: Area and estimated population for Southwark's three CIZs, the control region and the London Borough of Southwark

Supplementary Table 2: Number of licence applications and percentage issued in each CIZ and the control group by financial year. Data are stratified by outlet type (five categories).

Acknowledgements

The authors would like to acknowledge Southwark Council's Licensing Authority for providing access to the data for this study and for providing valuable context surrounding the local licensing policy.

Funding

This work was supported by the National Institute for Health Research (NIHR) under the Collaborations for Leadership in Applied Health Research and Care (CLAHRC) programme for North West London. The views expressed in this publication are those of the author(s) and not necessarily those of the NHS, the NIHR or the Department of Health.

Conflict of Interest

None.

Contributors

CS completed the literature review for this study, led on the data analysis and drafted the manuscript. DF provided context as to the local licensing policy. HW supported the development of the statistical model. CW created figure 1 and sourced the data for Supplementary Table 1. RP critically reviewed the manuscript and revised a second draft. HW, AP and RP inputted into the data interpretation and revised the final manuscript. All authors have approved the final manuscript.

For peer review only

For peer review only - http://bmjopen.bmj.com/site/about/guidelines.xhtml

Data Sharing Statement

No additional data are available.

For peer review only

For peer review only - http://bmjopen.bmj.com/site/about/guidelines.xhtml

Reference List

[1] Barbor T, Caetano R, Casswell S, et al, Alcohol: no ordinary commodity - research and public policy, Oxofrd Unversity Press, 2003.

[2] NHS Digital, Health Survey for England, 2016. [Available from: www.digital.nhs.uk], 2017.

[3] Burton R, Henn C, Lavoie D, O'Connor R, Perkins C, Sweeney K, Greaves F, Ferguson B, Beynon C, Belloni A, Musto V. The public health burden of alcohol and the effectiveness and cost-effectiveness of alcohol control policies: an evidence review. 2016.

[4] Campbell CA, Hahn RA, Elder R, Brewer R, Chattopadhyay S, Fielding J, Naimi TS, Toomey T, Lawrence B, Middleton JC, Task Force on Community Preventive Services. The effectiveness of limiting alcohol outlet density as a means of reducing excessive alcohol consumption and alcohol-related harms. American journal of preventive medicine. 2009 ;37(6):556-69.

[5] Jackson R, Johnson M, Campbell F, Messina J, Guillaume L, Meier PS, Goyder E, Chilcott J, Payne N. Interventions on control of alcohol price, promotion and availability for prevention of alcohol use disorders in adults and young people. London: The University of Sheffield, for NICE Centre for Public Health Excellence. 2010

[6] Stockwell T, Gruenewald PJ. Controls on the physical availability of alcohol. The essential handbook of treatment and prevention of alcohol problems. 2004 Feb 6:213-33.

[7] Institute of Alcohol Studies. Alcohol consumption factsheet 2013. [Accessed 09/08/2016]. Available from: http://www.ias.org.uk/.

[8] Richardson EA, Shortt NK, Pearce J, Mitchell R. Alcohol-related illness and deaths in Scottish neighbourhoods: is there a relationship with the number of alcohol outlets. Edinburgh: Centre for Research on Environment, Society and Health and Alcohol Focus Scotland. 2014 Oct 2..

For peer review only

For peer review only - http://bmjopen.bmj.com/site/about/guidelines.xhtml

[9] Gmel G, Holmes J, Studer J. Are alcohol outlet densities strongly associated with alcohol-related outcomes? A critical review of recent evidence. Drug and alcohol review. 2016 Jan;35(1):40-54.

[10] Morrison C, Cerdá M, Gorman DM, et al. Commentary on Gmel et al. (2015): Are alcohol outlet densities strongly associated with alcohol-related outcomes? A critical review of recent evidence. Drug Alcohol Rev. 2015;35(1):55–57.

[11] Licensing Act 2003. England and Wales. London: HMSO 2003

[12] Sharpe CA, Poots AJ, Watt H, Franklin D, Pinder RJ. Controlling alcohol availability through local policy: an observational study to evaluate Cumulative Impact Zones in a London borough. Journal of Public Health. 2017 Dec 11.

[13] Home Office. Revised Guidance issued under section 182 of the Licensing Act 2003. London: HMSO 2015

[14] Home Office. Alcohol and late night refreshment licensing England and Wales. National Statistics 2016.

[15] Humphreys DK, Smith DM. Alcohol licensing data: why is it an underused resource in public health?. Health & place. 2013 Nov 1;24:110-4.

[16] Egan M, Brennan A, Buykx P, De Vocht F, Gavens L, Grace D, Halliday E, Hickman M, Holt V, Mooney JD, Lock K. Local policies to tackle a national problem: Comparative qualitative case studies of an English local authority alcohol availability intervention. Health & place. 2016 Sep 1;41:11-8.

[17] Greater London Authority, Culture and the night-time economy. London, GLA, 2017.

[18] C. Williamson, Overview of Southwark's Population. Southwark's JSNA. Southwark Council: London. 2018.

[19] Civica. Civica Community Protection Overview 2016. [Accessed 23/07/2016]. Available from: https://www.civica.co.uk/public-protection/overview.

[20] Pitney Bowes. Data Sheet: MapInfo Location Intelligence [Available from: www.mapinfo.com Accessed 21/07/2016.

[21] Bland M. An introduction to medical statistics. Oxford University Press (UK); 2015.

For peer review only

For peer review only - http://bmjopen.bmj.com/site/about/guidelines.xhtml

[22] Stata Statistical Software [program]. vol. 13 version. College Station: TX:, 13 version. College Station: TX:.

For peer review only

For peer review only - http://bmjopen.bmj.com/site/about/guidelines.xhtml

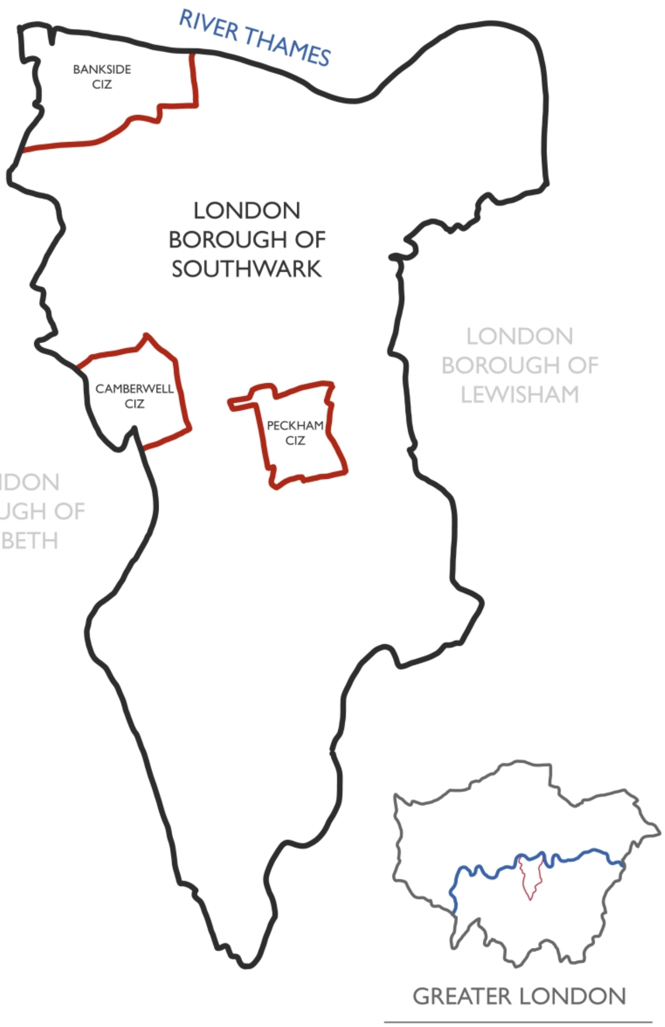

London Borough of Southwark’s three CIZs (inset: Map of the 33 London boroughs with the London Borough of Southwark highlighted).

755x1069mm (100 x 100 DPI)

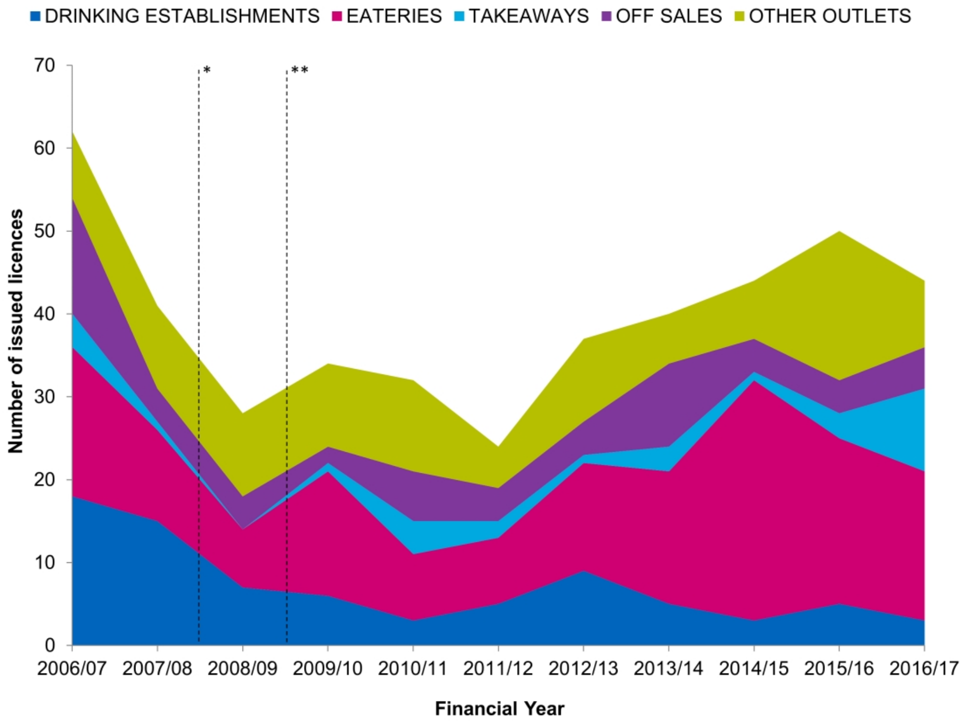

Figure 2: Number of issued licences per financial year in CIZ areas, by outlet type.
*Date of CIZ establishment, Peckham and Camberwell.
**Date of CIZ establishment, Bankside.

Supplementary Material

Supplementary Table 1: Area and estimated population for Southwark's three CIZs, the control region and the London Borough of Southwark

	AREA (km^2)	ESTIMATED TOTAL POPULATION (2016)**	
		Minimum	Maximum
**Bankside**	1.69	15,214	16,018
**Peckham**	0.91	11,226	13,820
**Camberwell**	0.74	6,518	8,474
**Control***	26.47	273,343	278,697
**Southwark**	29.81	311,655	

*The control region relates to the area of Southwark that is not a CIZ
**Southwark's CIZs are non-standard geographies: their boundaries do not align with Output Areas (OA). Output Areas are the lowest level of geography produced across all census topics. The average population in England and Wales on 27 March 2011 was 309 in OAs. Therefore, each CIZ population can only be estimated. The minimum population estimate relates the total population of all OAs that fully fit within the CIZ boundary. The maximum estimate relates the to the total population of all the OAs that fall fully within the CIZ boundary as well as those that are partially within the CIZ boundary. [1]

[1] Office for National Statistics, 2011 Census: Population and Household Estimates for Small Areas in England and Wales, March 2011, [Available from www.ons.gov.uk], 2012

For peer review only

Supplementary Table 2: Number of licence applications and percentage issued in each CIZ and the control group by financial year. Data are stratified by outlet type (five categories).

	2006/07	2007/08	2008/09	2009/10	2010/11	2011/12	2012/13	2013/14	2014/15	2015/16	2016/17	Total
**DRINKING ESTABLISHMENTS**												
**All CIZs**	**21**	**17**	**7**	**6**	**4**	**5**	**11**	**5**	**3**	**5**	**5**	**89**
% Issued	85.7	88.2	100	100	75.0	100	81.8	100	100	100	60.0	88.8
Bankside	**11**	**11**	**5**	**4**	**4**	**5**	**10**	**3**	**2**	**5**	**3**	**63**
% Issued	100	90.9	100	100	75.0	100	80.0	100	100	100	66.7	92.1
Peckham	**3**	**3**	**2**	**2**	**0**	**0**	**1**	**2**	**1**	**0**	**2**	**16**
% Issued	100	100	100	100	-	-	100	100	100	-	50.0	93.8
Camberwell	**7**	**3**	**0**	**0**	**0**	**0**	**0**	**0**	**0**	**0**	**0**	**10**
% Issued	57.1	66.7	-	-	-	-	-	-	-	-	-	60.0
**Control**	**47**	**19**	**11**	**3**	**6**	**8**	**6**	**6**	**15**	**7**	**20**	**148**
% Issued	91.5	73.7	81.8	66.7	83.3	75.0	100	83.3	80.0	85.7	85.0	84.5
**EATERIES**												
**All CIZs**	**18**	**12**	**9**	**15**	**8**	**8**	**14**	**18**	**29**	**21**	**20**	**172**
% Issued	100	91.7	77.8	100	100	100	92.9	88.9	100	95.2	90.0	94.8
Bankside	**15**	**9**	**6**	**10**	**4**	**6**	**9**	**9**	**17**	**16**	**13**	**114**
% Issued	100	88.9	83.3	100	100	100	88.9	88.9	100	93.8	84.6	93.9
Peckham	**1**	**0**	**2**	**3**	**2**	**1**	**5**	**5**	**7**	**5**	**3**	**34**
% Issued	100	0.0	50.0	100	100	100	100	80.0	100	100	100.0	94.1
Camberwell	**2**	**3**	**1**	**2**	**2**	**1**	**0**	**4**	**5**	**0**	**4**	**24**
% Issued	100	100	100	100	100	100	-	100	100	-	100	100
**Control**	**49**	**17**	**15**	**14**	**18**	**19**	**13**	**21**	**19**	**31**	**39**	**255**
% Issued	93.9	94.1	93.3	85.7	94.4	100	100	85.7	84.2	77.4	94.9	91.0
**TAKEAWAYS**												
**All CIZs**	**4**	**1**	**0**	**2**	**4**	**2**	**1**	**3**	**1**	**3**	**11**	**32**
% Issued	100	100	-	50.0	100	100	100	100	100	100	90.9	93.8
Bankside	**3**	**0**	**0**	**1**	**3**	**2**	**1**	**0**	**0**	**2**	**6**	**18**
% Issued	100	-	-	100	100	100	100	-	-	100	100	100
Peckham	**0**	**1**	**0**	**1**	**0**	**0**	**0**	**1**	**1**	**1**	**4**	**9**
% Issued	-	100	-	0	-	-	-	100	100	100	75.0	77.8
Camberwell	**1**	**0**	**0**	**0**	**1**	**0**	**0**	**2**	**0**	**0**	**1**	**5**
% Issued	100	-	-	-	100	-	-	100	-	-	100	100
**Control**	**19**	**6**	**2**	**1**	**1**	**6**	**1**	**4**	**3**	**5**	**7**	**55**
% Issued	100	83.3	100	100	100	66.7	100	100	100	100	71.4	90.9
**OFF-SALES**												
**All CIZs**	**14**	**5**	**6**	**2**	**7**	**6**	**4**	**11**	**4**	**4**	**5**	**68**
% Issued	100	80.0	66.7	100	85.7	66.7	100	90.9	100	100	100	89.7
Bankside	**7**	**3**	**5**	**1**	**5**	**1**	**1**	**5**	**4**	**3**	**4**	**39**
% Issued	100	100	60.0	100	100	100	100	100	100	100	100	94.9
Peckham	**5**	**1**	**0**	**1**	**2**	**2**	**3**	**2**	**0**	**1**	**1**	**18**
% Issued	100.0	-	-	100	50.0	-	100	50.0	-	100	100	72.2
Camberwell	**2**	**1**	**1**	**0**	**0**	**3**	**0**	**4**	**0**	**0**	**0**	**11**
% Issued	100	100	100	-	-	100	-	100	-	-	-	100

For peer review only

**Control**	**40**	**18**	**15**	**11**	**9**	**17**	**10**	**18**	**16**	**16**	**10**	**180**
% Issued	92.5	94.4	86.7	81.8	100	100	100	100	81.3	68.8	70.0	89.4
						**OTHER OUTLETS**						
**All CIZs**	**9**	**10**	**12**	**12**	**12**	**6**	**10**	**9**	**8**	**21**	**11**	**120**
% Issued	88.9	100	83.3	83.3	91.7	83.3	100	66.7	87.5	85.7	72.7	85.8
Bankside	**7**	**10**	**10**	**12**	**9**	**5**	**7**	**6**	**7**	**15**	**9**	**97**
% Issued	85.7	100	90.0	83.3	100	80.0	100	66.7	100	80.0	77.8	87.6
Peckham	**1**	**0**	**2**	**0**	**2**	**1**	**3**	**3**	**0**	**5**	**1**	**18**
% Issued	100	-	50.0	0.0	50.0	100	100	66.7	-	100	0.0	77.8
Camberwell	**1**	**0**	**0**	**0**	**1**	**0**	**0**	**0**	**1**	**1**	**1**	**5**
% Issued	100	-	-	-	100	-	-	-	0.0	100	100	80.0
**Control**	**18**	**6**	**5**	**12**	**8**	**10**	**10**	**11**	**19**	**20**	**16**	**135**
% Issued	94.4	100	60.0	83.3	100	100	100	100	78.9	80.0	62.5	85.9

For peer review only

STROBE 2007 (v4) checklist of items to be included in reports of observational studies in epidemiology*

Checklist for cohort, case-control, and cross-sectional studies (combined)

Section/Topic	Item #	Recommendation	Reported on page
**Title and abstract**	1	(*a*) Indicate the study's design with a commonly used term in the title or the abstract	1
		(*b*) Provide in the abstract an informative and balanced summary of what was done and what was found	2
**Introduction**			
Background/rationale	2	Explain the scientific background and rationale for the investigation being reported	4-6
Objectives	3	State specific objectives, including any pre-specified hypotheses	5
**Methods**			
Study design	4	Present key elements of study design early in the paper	2
Setting	5	Describe the setting, locations, and relevant dates, including periods of recruitment, exposure, follow-up, and data collection	2 and 7
Participants	6	(*a*) *Cohort study*—Give the eligibility criteria, and the sources and methods of selection of participants. Describe methods of follow-up<br>*Case-control study*—Give the eligibility criteria, and the sources and methods of case ascertainment and control selection. Give the rationale for the choice of cases and controls<br>*Cross-sectional study*—Give the eligibility criteria, and the sources and methods of selection of participants	Data source is explained on page (this is data that wa readily available)
		(*b*) *Cohort study*—For matched studies, give matching criteria and number of exposed and unexposed<br>*Case-control study*—For matched studies, give matching criteria and the number of controls per case	N/A
Variables	7	Clearly define all outcomes, exposures, predictors, potential confounders, and effect modifiers. Give diagnostic criteria, if applicable	7
Data sources/ measurement	8*	For each variable of interest, give sources of data and details of methods of assessment (measurement). Describe comparability of assessment methods if there is more than one group	7
Bias	9	Describe any efforts to address potential sources of bias	7 (two adjustmen variables were included in the statistical model)
Study size	10	Explain how the study size was arrived at	7 (all licence applications within defined time perio were included, a study sample was therefore not

For peer review only

			necessary)
Quantitative variables	11	Explain how quantitative variables were handled in the analyses. If applicable, describe which groupings were chosen and why	7
Statistical methods	12	(*a*) Describe all statistical methods, including those used to control for confounding	7
		(*b*) Describe any methods used to examine subgroups and interactions	7
		(*c*) Explain how missing data were addressed	N/A (a comprehensive dat set was sourced)
		(*d*) *Cohort study*—If applicable, explain how loss to follow-up was addressed<br>*Case-control study*—If applicable, explain how matching of cases and controls was addressed<br>*Cross-sectional study*—If applicable, describe analytical methods taking account of sampling strategy	N/A
		(*e*) Describe any sensitivity analyses	N/A
**Results**			
Participants	13*	(a) Report numbers of individuals at each stage of study—eg numbers potentially eligible, examined for eligibility, confirmed eligible, included in the study, completing follow-up, and analysed	N/A
		(b) Give reasons for non-participation at each stage	N/A
		(c) Consider use of a flow diagram	N/A
Descriptive data	14*	(a) Give characteristics of study participants (eg demographic, clinical, social) and information on exposures and potential confounders	N/A
		(b) Indicate number of participants with missing data for each variable of interest	7
		(c) *Cohort study*—Summarise follow-up time (eg, average and total amount)	N/A
Outcome data	15*	*Cohort study*—Report numbers of outcome events or summary measures over time	10, 11-12 (see als supplementary material)
		Case-control study—Report numbers in each exposure category, or summary measures of exposure	10, 11-12 (see als supplementary material)
		Cross-sectional study—Report numbers of outcome events or summary measures	10, 11-12 (see als supplementary material)
Main results	16	(*a*) Give unadjusted estimates and, if applicable, confounder-adjusted estimates and their precision (eg, 95% confidence interval). Make clear which confounders were adjusted for and why they were included	11-12
		(*b*) Report category boundaries when continuous variables were categorized	N/A
		(*c*) If relevant, consider translating estimates of relative risk into absolute risk for a meaningful time period	N/A

For peer review only

Other analyses	17	Report other analyses done—eg analyses of subgroups and interactions, and sensitivity analyses	10, 11-12
**Discussion**			
Key results	18	Summarise key results with reference to study objectives	13
Limitations	19	Discuss limitations of the study, taking into account sources of potential bias or imprecision. Discuss both direction and magnitude of any potential bias	13-14
Interpretation	20	Give a cautious overall interpretation of results considering objectives, limitations, multiplicity of analyses, results from similar studies, and other relevant evidence	13-15
Generalisability	21	Discuss the generalisability (external validity) of the study results	15
**Other information**			
Funding	22	Give the source of funding and the role of the funders for the present study and, if applicable, for the original study on which the present article is based	16

*Give information separately for cases and controls in case-control studies and, if applicable, for exposed and unexposed groups in cohort and cross-sectional studies.
Note: An Explanation and Elaboration article discusses each checklist item and gives methodological background and published examples of transparent reporting. The STROBE checklist is best used in conjunction with this article (freely available on the Web sites of PLoS Medicine at http://www.plosmedicine.org/, Annals of Internal Medicine at http://www.annals.org/, and Epidemiology at http://www.epidem.com/). Information on the STROBE Initiative is available at www.strobe-statement.org.

For peer review only
